# Children from six cultures reliably help their peers—but in different ways

**DOI:** 10.1073/pnas.2515233123

**Published:** 2026-10-02

**Authors:** Henriette Zeidler, Marie Schäfer, Esther Herrmann, Patricia Kanngiesser, Luke Maurits, Daniel Haun, Michael Tomasello

**Affiliations:** ^a^Department of Dynamic and Clinical Psychology and Health, Sapienza University, Rome 00185, Italy; ^b^ Wilhelm Wundt Institute for Psychology, Leipzig University, Leipzig 04109, Germany; ^c^ School of Psychology, Sport and Health Sciences, University of Portsmouth, Portsmouth PO1 2DY, United Kingdom; ^d^ School of Psychology, University of Plymouth, Plymouth PL4 8AA, United Kingdom; ^e^ Department of Comparative Cultural Psychology, Max Planck Institute for Evolutionary Anthropology, Leipzig 04103, Germany; ^f^ Department of Psychology and Neuroscience, Duke University, Durham, NC 27708

**Keywords:** helping, prosocial behavior, children, cross-cultural

## Abstract

Helping others is an integral part of human sociality, but systematic cross-cultural comparisons of children’s helping behavior are rare. Existing work has mainly focused on interactions between adults and children, paying less attention to peer dynamics—which are more relevant in many cultural settings. Here, we report a study of 4- to 9-y-old children’s (N = 336) spontaneous helping in 6 highly diverse populations in Latin America, Africa, and Europe (Wichí, Quechua, ≠Akhoe Hai||om, Samburu, Kikuyu, and Germans). In all settings, we observed pairs of same-aged children in an experimental task in which one partner could assist the other by providing either instrumental help or information. Across cultures, children reliably helped their peers by offering either type of help, with the provision of information increasing over age. We found striking commonalities in peer helping in all settings, with only small culture-specific patterns in children’s responses. This suggests that helping among children is a universal human behavior which is subtly shaped by local norms and practices.

## Significance

All over the world people routinely help one another. Helping provides valuable opportunities to affiliate with others—the foundation of human sociality. Focusing on peer interactions, we tested 4- to 9-y-old children in an experimental task and found striking similarities and matching developmental trends across a wide range of settings: Children everywhere reliably helped their partners by providing information or instrumental help, and with age increasingly chose the most salient strategy. The only culture-specific behavior was supplying information and instrumental help at the same time, which happened more frequently in our urban, industrialized sample as compared to our rural, subsistence-based communities. Our findings suggest that peer helping is a universal behavior that is subtly shaped by local norms.

All over the world, across many diverse cultures, people routinely help one another in their daily activities. Helping provides valuable opportunities to affiliate with others—the foundation of human sociality. It is therefore not surprising that helping emerges early in ontogeny and across different contexts (1–3). In rural Canada, India, and Peru infants as young as 18 mo provide instrumental assistance by helping adults to complete a task (4); in the US toddlers help around the home (5); and in Germany, Brazil, and India young children assist their mothers in a variety of situations (6–8). While both experimental studies and anthropological work suggest an early, universal proclivity to help, there is also strong evidence for the impact of culture in shaping children’s helping behaviors from early on.

For one, parents from different cultures embrace different socialization goals and practices, and depending on these goals, children seem to develop different motivations to help. For example, while observations of mother–infant interactions confirmed that helping was grounded in empathic concern for others in Germany, they also showed that relational socialization goals, especially obedience, were stronger predictors of toddlers’ prosocial behaviors in India (9). Along the same lines, toddlers’ helping behaviors were related to different types of maternal scaffolding: asking, pleading, and giving explanations in Germany; and serious and insistent requesting in Brazil (6). Different socialization practices may also lead to differences in children’s overall tendency to help. In a recent study (8), Indian toddlers helped more than German toddlers, but also had more opportunities to help around the home. Culture-specific predictors of helping behaviors were also found in a study with 18-mo-old British and Ugandan children: In the United Kingdom, more maternal scaffolding strategies, and in Uganda, greater alignment with relational socialization goals positively predicted the likelihood of infant helping (10).

For another, different socioeconomic settings provide different opportunities for children to help and thus influence in which situations they can practice their helping skills. In many parts of the world, children as young as 14 mo start assisting others around the home (11). Participating in everyday activities allows them to socialize and learn and prepares them for a life of collaborative, community-based work (3, 12). The availability of parents and older peers as role models, and the wide range of tools and small tasks to practice with create an environment that supports the nurturance of increasingly skillful helpers (13). While children in rural, subsistence-based societies do indeed make valuable contributions to their families’ subsistence by fetching water, collecting firewood, preparing food, running errands, and taking care of younger siblings (14–18), the active help provided by young children may be less valuable in urban, industrialized societies where parents typically work outside the home and childcare is institutionalized (19). As a consequence, children’s helping behaviors might differ later in life (3).

While these studies underline the universal existence of early helping behaviors, they also provide strong evidence for the impact of culture in shaping them. Opportunities to help adults may be particularly variable across cultures and dependent on the socioeconomic context, whereas helping peers may be important everywhere. Whether with or without adult supervision, children spend large amounts of time in peer groups (20, 21), and a lot of learning—and helping—occurs in these contexts. Forager children, for instance, learn to produce a substantial amount of their own calories through peer-teaching, which is even more frequent than adult-led teaching (22). Peer group activities offer unique opportunities for exploration and learning and give rise to distinct peer cultures that can act as reservoirs for diverse community knowledge (23).

Peers may thus play an important role in shaping the development of prosocial and normative behaviors (24)—especially at a time when additional factors such as moral emotions, perspective-taking abilities, and reputational concerns come into play (25–27). Additionally, prosocial behaviors also change as children become aware of the costs and benefits of their actions. When helping adults comes at a relatively low cost, children help at similar ages and levels across cultures (28). Costly helping, on the other hand, may depend on children’s early culture-specific experiences (29). Thus, with increasing age, children’s helping behaviors may become more adjusted to the specific social expectations, norms, and values of their cultural environment and therefore increasingly vary across societies.

In the current study, therefore, we investigated 4- to 9-y-old children’s tendency to help their peers in six culturally diverse populations from Europe, Africa, and South America: a typical large-scale, Western society (Germany), settled foragers (Wichí in Argentina, and ≠Akhoe Hai||om in Namibia), pastoralists (Samburu in Kenya), and small-scale farmers (Quechua in Bolivia, and Kikuyu in Kenya). Our sample thus not only includes urban, industrialized and rural, subsistence-based societies but also covers differences in the amount of help children are expected to provide—from very few obligations to help in Germany and the two foraging groups to major contributions to domestic chores in the farming and pastoralist contexts. In addition, our settings differed greatly in the amount of time children spend without being supervised by adults. Whereas German children are rarely on their own, children in the other settings regularly spend time in peer groups away from adult supervision. Finally, our sample also differed in the type of instructions children would typically receive. While all children in our study were enrolled in school, attendance varied greatly (Table 1). German children rarely missed a day of school, and Kikuyu children would also be present most of the time. Quechua and Wichí children attended school fairly regularly as well. Hai//om children often chose to engage in other activities instead, and Samburu children frequently missed school to look after livestock or siblings. The largest differences in children’s learning experiences, however, occurred outside school. While children in small-scale settings mostly learn through observation and pitching in, German parents tend to provide detailed verbal instructions and explanations (30).

**Table 1.** Overview of study participants

Population	Location	Social organization	Economic base	Number of children per family	School enrolment (county level)	N (female)	M Age (SD)
≠Akhoe Hai||om	Namibia, Africa	Village	Hunter-gatherers, some wage work	4.1	67%	56 (28)	6.6 (1.53)
Germans	Germany, Europe	Town	Wage work	1.5	100%	60 (31)	6.2 (1.68)
Kikuyu	Kenya, Africa	Village	Agriculture, wage work	2.7	94%	58 (29)	6.4 (1.73)
Samburu	Kenya, Africa	Village	Herding, some wage work	6.3	57%	68 (30)	6.5 (1.65)
Quechua	Bolivia, South America	Village	Agriculture, wage work	3.5	83%	47 (24)	6.4 (1.65)
Wichí	Argentina, South America	Village	Craft production, some wage work	4.5	87%	47 (23)	6.2 (1.61)
All						336 (165)	6.4

**Fig. 1.**
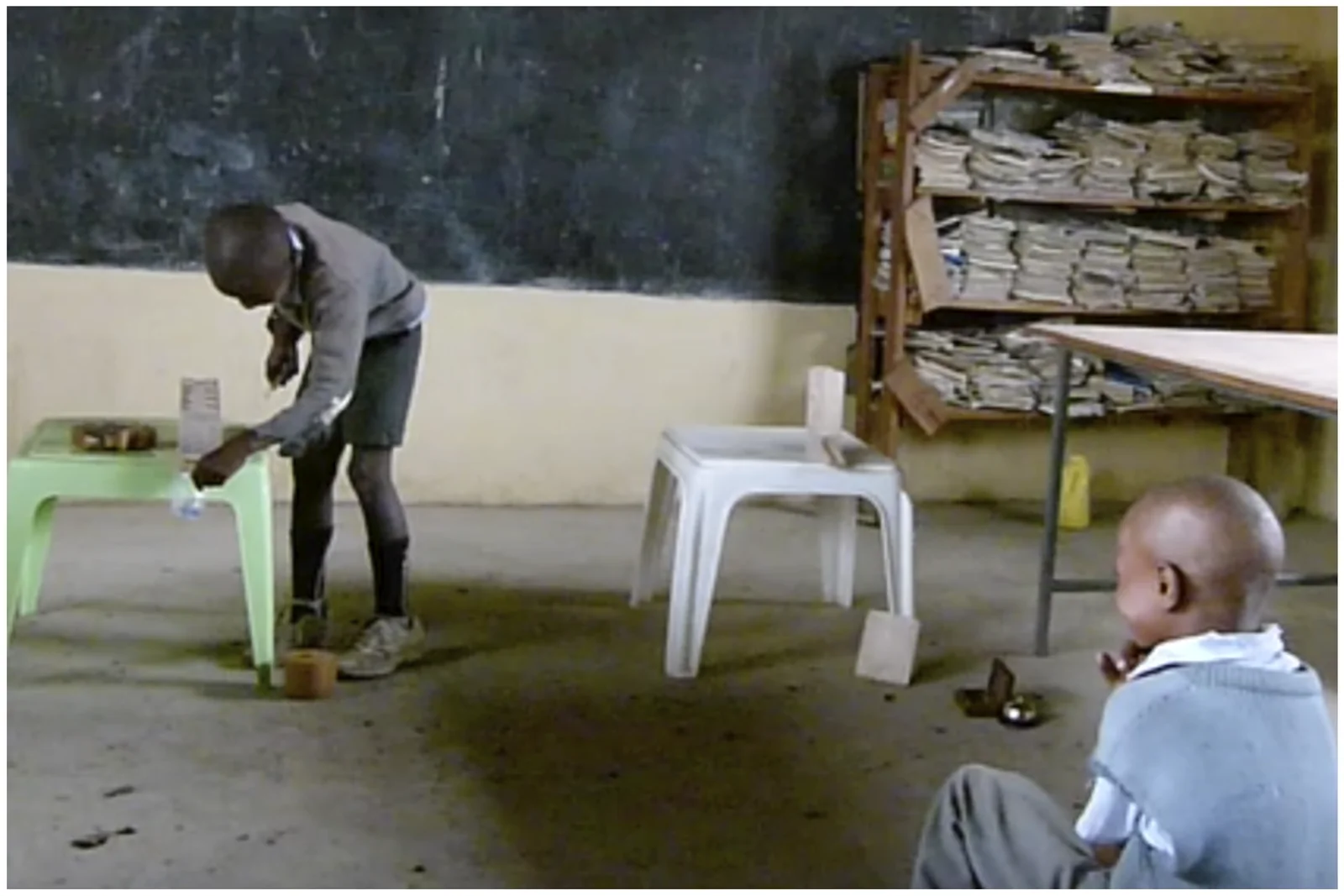
Experimental setup.

To tap into children’s natural proclivity to help, we designed a playful interaction which would be intuitive across our settings and not viewed as a chore, and paired participants with a same-aged partner to avoid potential asymmetries in knowledge and status. In all settings, we observed two peers in a standardized task which provided one of the children with various opportunities to assist the other. Specifically, the (potential) helper watched from a few meters away as their peer played a marble game and encountered two types of problems: In one case, a container holding the marbles broke and its contents spilled on the floor; and in another case, a box would not open. The observer had played the same game previously and therefore knew that the problem could be solved either by offering information or by providing instrumental help through actively solving the task for the peer (Fig. 1).

**Fig. 2.**
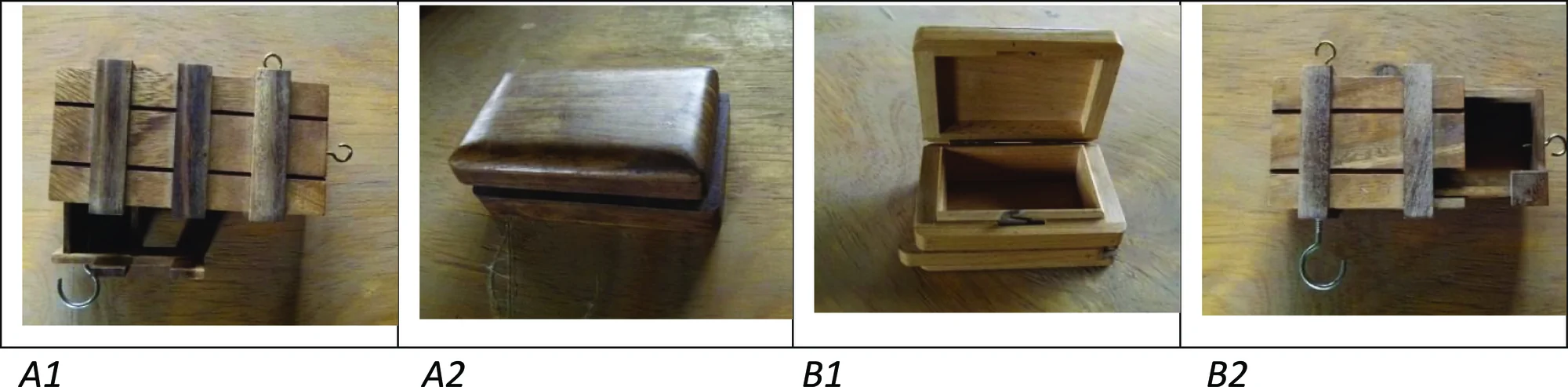
Boxes used in the game. Half of the children learned how to open boxes *A1* and *A2*, and the other half learned how to open boxes *B1* and *B2*. In the test, children from group A needed to open boxes *B1* and *B2* and vice versa, creating a chance for their knowledgeable peers from the other group to help.

While both types of help were useful in each case, instrumental help was more salient for one problem (collecting marbles), and providing information was more relevant for the other (explaining how to open a specific box, see Fig. 2). In addition, peers could have easily picked up the marbles themselves but were clearly struggling with the box and thus in more need of help.

We were interested, first of all, in whether children from different cultural backgrounds would help one another at similar rates across societies and across ages. Our second question was if, with increasing age, children would adapt their helping behaviors to the partner’s needs and help more when the other was clearly struggling. Finally, we wanted to see whether children’s ways of helping (i.e., through instrumental help and informing) would differ between societies and change across age. In order to capture these variations, we focused on children between 4 and 9 y of age, which is when a wide range of prosocial behaviors undergo significant changes and children become increasingly attuned to the norms of their respective societies (26, 31).

Given that previous studies had found similarities in young children’s prosocial behaviors and an increasing number of differences later on (32), we were expecting to see similar rates and types of helping at the lower end of our age range, and more nuanced responses in older children—which is also when more strategic behaviors have been shown to emerge (27), especially when prosocial behaviors come at a cost (33). Helping in our study was relatively low cost in all situations, but we expected children to help more overall with opening boxes since they presented a more difficult challenge than picking up marbles and thus created a greater need for help. While both informative and instrumental help were possible responses to each of the problems, we expected to find differences in helping styles based on children’s cultural backgrounds. In this sense, German children, who typically receive more verbal instructions from adults (30), should provide more informative help than other children who tend to learn through actively participating in everyday tasks.

## Results

The main model clearly outperformed the other two models in the Widely Applicable Information Criterion (WAIC) comparison, with estimated differences in predictive accuracy exceeding two SE (full model vs. intercept-only model: ∆ELPD = −46.25, SE = 9.67; full model vs. no-country-effects model: ∆ELPDFULLvsNOCOUNTRY = −72.84, SE = 13.59). This suggests that both systematic effects of our predictor variables and cross-cultural variation in behavior are necessary to account for the data. We therefore report results only for the full model. The full set of model parameter estimates is presented in *SI Appendix*, but for ease of interpretation, we present results here primarily in terms of the model’s predicted probabilities of different helping responses for different accidents and demographics, as well as contrasts in these probabilities.

### Overall Helping Rates.

We begin by considering the overall tendency to help across societies in the two helping contexts (box and marble accidents). We do so by summing the estimated probabilities of providing informative help, instrumental help, or both together to arrive at a single probability of helping, and at the same time marginalizing out sex effects, and marginalizing over any developmental effects by combining posterior predictive probabilities for children at ages 4, 5, 6, 7, 8, and 9. These helping probability estimates are shown in Table 2.

**Table 2.** Estimated probabilities of displaying any kind of helping across cultures and accident types, marginalizing out developmental effects (posterior mean, 95% HPD interval, and posterior probability that helping probability is above 0.5)

Culture	Marbles	Box
≠Akhoe Hai||om	0.70 (0.62 to 0.78, 1.00)	0.85 (0.80 to 0.90, 1.00)
German	0.62 (0.54 to 0.69, 0.86)	0.85 (0.79 to 0.89, 1.00)
Kikuyu	0.45 (0.38 to 0.53, 0.12)	0.75 (0.68 to 0.81, 1.00)
Quechua	0.76 (0.68 to 0.82, 1.00)	0.88 (0.84 to 0.92, 1.00)
Samburu	0.61 (0.53 to 0.67, 1.00)	0.81 (0.76 to 0.86, 1.00)
Wichí	0.67 (0.59 to 0.75, 1.00)	0.84 (0.79 to 0.89, 1.00)

In general, children were clearly helpful. Posterior mean estimates of the probability of offering some kind of help were above 0.5 (ranging from 0.6 to 0.88) for both accident types in all cultures with the single exception of Kikuyu children, whose probability of helping in response to marble accidents was 0.45. The estimated probability of helping in response to box accidents (the situation in which help was necessary for the peer to succeed) was higher than in response to marble accidents (the situation in which help was not necessary for the peer to succeed) in every culture. In fact, for box accidents the 95% HPD interval for the helping probability was entirely above 0.5 in every culture, whereas this was not always the case for marble helping probabilities. However, with the exception of Kikuyu children, evidence was still strong that helping was the more probable response overall, with the posterior probability of helping being more likely than not helping never falling below 0.82. Thus, confirming our hypotheses, children across societies were very likely to help, and helped one another at similar rates—in particular in the situation in which the partner was in greater need of help (i.e., opening a box). In the situation in which the partner could solve the problem (i.e., picking up marbles) quite easily without help, children across societies were less likely to help and varied more in their responses.

### Development of Helping Rates.

Next, we consider broad developmental trends in helping rates by retaining the focus on a single probability of providing any kind of help—regardless of modality, but considering how this probability changes with age for the two accident types.

**Fig. 3.**
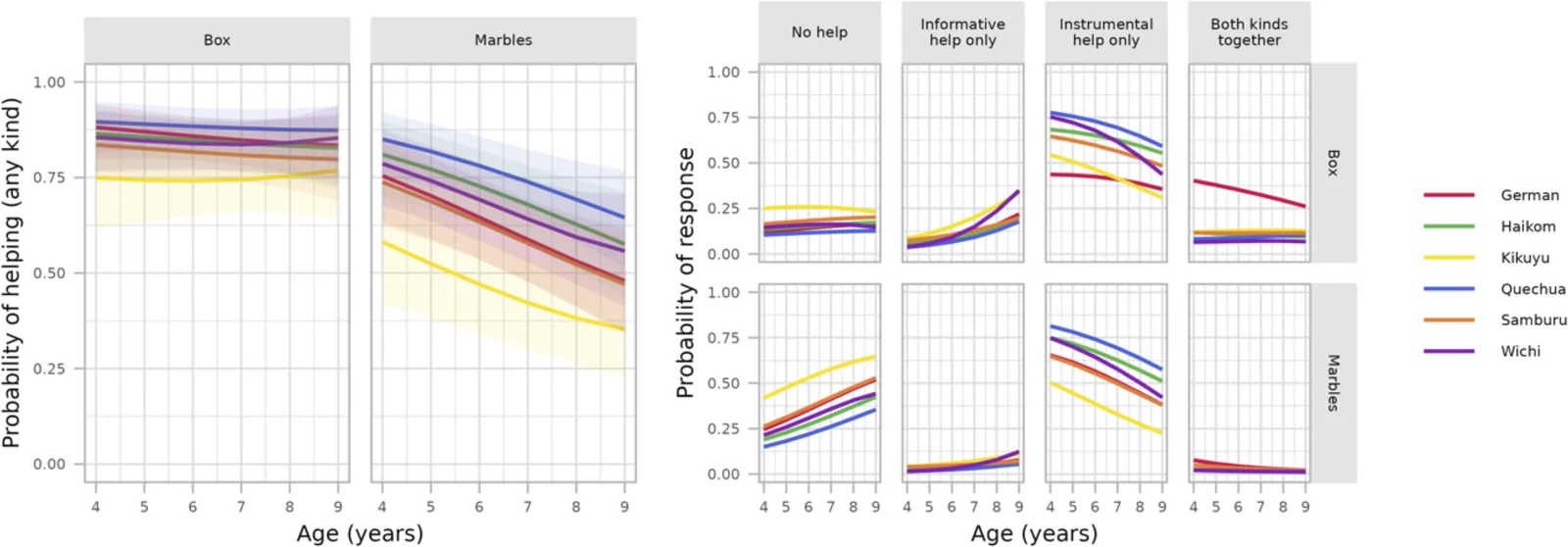
Developmental trajectory for types of help offered across cultures.

As visualized in the *Left* part of Fig. 3, the probability of helping in response to box accidents was very stable across the 4- to 9-y age range in all cultures. When considering posterior predictive contrasts in the helping probability between 4- and 9-y-olds, the 95% HPD interval did not exclude zero for any culture, and the posterior probability of the effect being in one direction was generally low. The only cultures where this posterior probability exceeded 0.75 were German and Hai||om children, who showed modest evidence of small positive effects, with posterior mean increases in helping probability of 0.05 and 0.04, respectively.

In contrast, the probability of helping in response to marble accidents clearly decreased with age in every culture, with each culture’s 95% HPD interval for the decrease in helping probability excluding zero. The posterior mean estimates for this decrease ranged from 0.20 to 0.27, and the 95% HPD intervals for the probability of 9-y-old children helping in response to marble accidents included 0.5 for every culture except the Quechua and Kikuyu.

Thus, in accordance with our expectations, children’s behavior becomes more discriminative with increasing age across societies, with helping ratings remaining stable or slightly increasing in situations in which more help is needed (opening boxes) and decreasing in situations in which help is not absolutely necessary (picking up marbles).

### Overall Rates of Different Types of Helping.

Finally, we look at how children helped their peers in each of the two situations, and how different helping behaviors develop in each society. Thus, we take a more fine-grained perspective by switching focus to separate probabilities for the three different helping responses (instrumental help, informative help, and a combination of both) and considering how their relative likelihood changes with age.

When marginalizing across age, instrumental helping alone was by far the most frequent response to marble accidents, i.e., children would pick up marbles for their partners (Table 3).

**Table 3.** Estimated probabilities of different helping responses to marble-type accidents across cultures (posterior mean and 95% HPD interval) when marginalizing out developmental trends

Culture	Info	Inst	Both
≠Akhoe Hai||om	0.04 (0.02 to 0.06)	0.64 (0.56 to 0.73)	0.02 (0.00 to 0.04)
German	0.04 (0.02 to 0.07)	0.53 (0.45 to 0.61)	0.04 (0.00 to 0.09)
Kikuyu	0.07 (0.04 to 0.10)	0.36 (0.28 to 0.44)	0.02 (0.01 to 0.05)
Quechua	0.03 (0.02 to 0.05)	0.71 (0.63 to 0.79)	0.01 (0.00 to 0.03)
Samburu	0.05 (0.03 to 0.08)	0.53 (0.44 to 0.60)	0.03 (0.01 to 0.06)
Wichí	0.05 (0.03 to 0.09)	0.60 (0.51 to 0.69)	0.01 (0.00 to 0.04)

In contrast, posterior mean estimates for the probability of providing informative help alone never exceeded 0.07 (for Kikuyu children), while those for providing both kinds of help together never exceeded 0.04 (for German children). There was no developmental shift in this tendency, with these other responses remaining very rare across the full 4- to 9-y-old age range.

Marginalizing out developmental trends, instrumental helping was also the most probable kind of help offered by children from all cultures in response to the box accident (Table 4), and was particularly frequently offered by Quechua children (posterior mean probability 0.71). While rarer, the other responses were not exceptionally unlikely either and depended on children’s cultural background. For example, German children were almost equally likely to offer both kinds of help simultaneously and did so at more than three times the rate of children from any other culture. Kikuyu children were not only least likely to offer any kind of help for box accidents (Table 4) but were also the most likely to offer informative help alone (posterior mean probability 0.19).

**Table 4.** Estimated probabilities of different helping responses to box-type accidents across cultures (posterior mean and 95% HPD interval) when marginalizing out developmental trends

Culture	Info	Inst	Both
≠Akhoe Hai||om	0.11 (0.07 to 0.15)	0.64 (0.54 to 0.71)	0.10 (0.06 to 0.17)
German	0.11 (0.07 to 0.16)	0.41 (0.33 to 0.49)	0.34 (0.25 to 0.43)
Kikuyu	0.19 (0.14 to 0.25)	0.43 (0.35 to 0.53)	0.12 (0.07 to 0.19)
Quechua	0.09 (0.06 to 0.13)	0.70 (0.62 to 0.78)	0.09 (0.05 to 0.15)
Samburu	0.12 (0.09 to 0.17)	0.58 (0.59 to 0.65)	0.11 (0.07 to 0.17)
Wichí	0.15 (0.10 to 0.21)	0.63 (0.54 to 0.71)	0.07 (0.03 to 0.12)

**Fig. 4.**
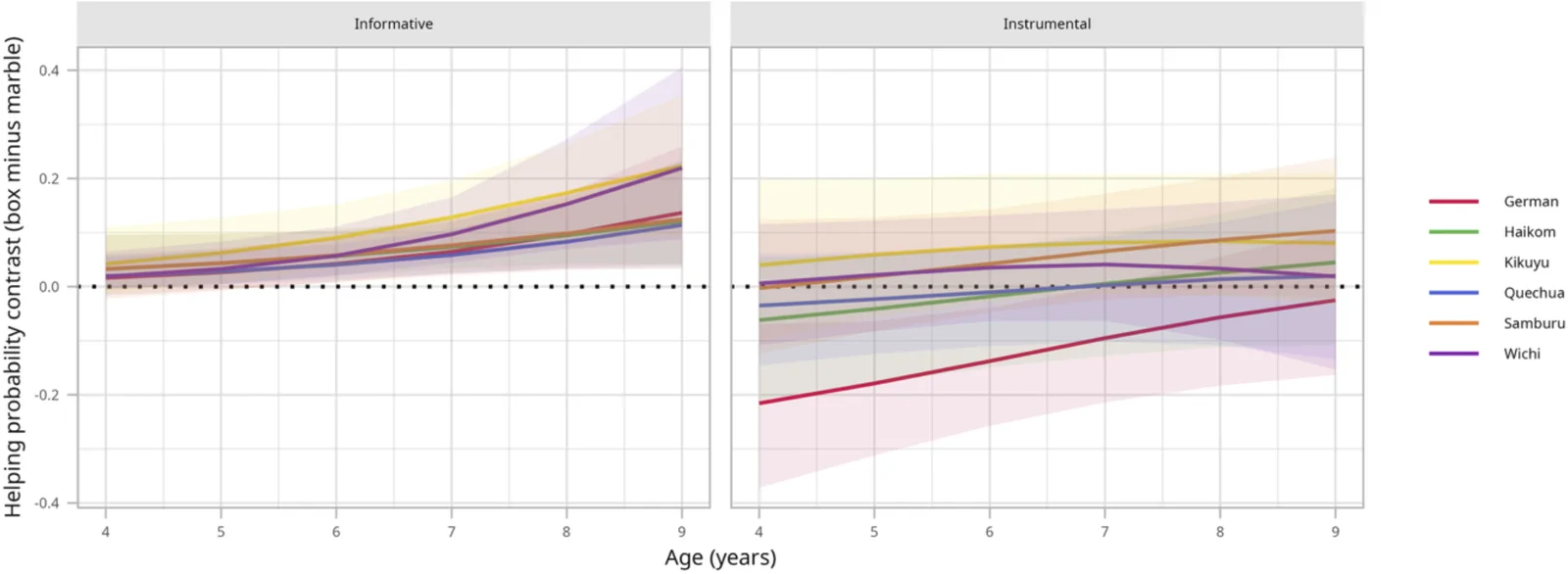
Interaction between accident type and age for the two types of helping (informative vs. instrumental), visualized as the difference in probabilities (posterior mean and 95% HPD interval) of offering the two kinds of help.

When looking at the interaction between accident type and age for the two types of helping separately, the difference in the probability of informative helping between box and marble accidents increases with age in all cultures, with the 95% HPD interval eventually (from about 5.5 y onward) excluding zero: Older children are clearly more likely to offer informative help only in response to box accidents than marble accidents. In contrast, for instrumental helping alone, the 95% HPD for the difference in probability between the two accident types includes zero at all ages for all cultures except German children, with young Germans being less likely to offer instrumental helping alone for box accidents than marble accidents, due to their cross-culturally unusual preference for helping in both modalities together (Fig. 4).

Thus, while instrumental help was the most common response overall, information (instead of or in addition to instrumental help) was more likely to be offered in response to box accidents as compared to marble accidents. In line with our expectations, children thus provided more information in cases where it was particularly useful, and the frequency of this behavior depended on culture.

### Development and Cross-Cultural Variation of Types of Help.

When considering the probability of providing any kind of help at all, the developmental trajectory for box accidents was seen to be flat, with no clear difference between 4- and 9-y-olds (*Left* panel in Fig. 3). However, when considering separate kinds of helping, a more nuanced picture emerged, as shown in the *Right* panel in Fig. 3. Generally speaking, younger children were much more likely to offer instrumental help alone. As children matured, they gradually became less likely to offer instrumental help, but at the same time more likely to offer informative help. Underneath the roughly constant probability of helping at all was a clear shift in the preferred form of helping. The extent of this shift in preference varied. In some populations, the oldest children were roughly equally likely to offer informative or instrumental help, but in most cases instrumental help remained more likely. The German participants were an exception to this overall trend: Young Germans were roughly equally likely to provide instrumental help alone or in combination with informative help. Their tendency to offer both kinds of help simultaneously decreased slightly with age but remained more likely than providing informative help alone. This was one of the starkest cross-cultural differences in the dataset: German children responding to box accidents were between three to four times more likely to offer both kinds of help than children from any other country. There was also evidence of another cross-cultural difference in developmental trends: the rate at which Wichí children increased their information-only responses with age was above the cross-cultural mean (see *Right* panel in Fig. 3).

Also independently of age, our data showed strong evidence of cross-cultural variation in the forms of helping behavior, with the 95% HPD intervals for several cultures’ random intercepts excluding zero. In particular, Kikuyu children were less likely to offer instrumental help than the population mean, while Quechua children were more likely to offer it. German children were more likely to offer a combination of informative and instrumental help than other children, while Wichí children’s behavior in response to box accidents changed more rapidly with age than other children’s. However, while the data provide strong support for the presence of all these cultural differences, the actual magnitude of these differences was relatively small, and none of the cultures could reasonably be considered an outlier with respect to the overall picture. Indeed, Fig. 3 clearly shows a striking similarity in behavior across all sampled cultures.

Our data did not provide strong support for influence of sex on helping behavior, with none of the sex-related model parameters having 95% HPD intervals excluding zero. There was modest support for some small interactions, and these are discussed in *SI Appendix*.

## Discussion

The current study revealed striking similarities in children’s peer helping behavior in a standardized task across a wide range of different cultural settings. Overall, children provided mainly instrumental help, but the amount of information provided increased, and instrumental helping declined with age. Again, this pattern was found in almost all settings, with only two notable differences: Kikuyu children were slightly less likely to provide instrumental help than anyone else, and German children were far more likely than others to provide information in addition to instrumental help. Interestingly, the types of help offered seem to match typical parenting behaviors in our settings: While children in rural, subsistence-based societies often learn by pitching in and rarely receive abstract verbal information on how to solve a task, middle-class parents in Germany and similar Global North settings often provide extensive verbal explanations along with practical demonstrations (1). Rather than just solving the problem by either informing or providing instrumental help, German children thus seemed to apply the type of helping behavior they would typically have experienced themselves.

In line with previous studies showing that children adjust their prosocial behaviors to situational demands as they grow older (34, 35), children in our sample also seemed to become increasingly aware of their peers’ needs and reacted accordingly. Children did not just help randomly but adapted their behaviors to the specific situations—and that was true everywhere. Children from all samples helped more when their partners were clearly struggling (i.e., trying to open a box) as compared to when they could have solved the task alone (by simply picking up items).

This study thus extends previous research on children’s helping by adding experimental data from peer settings across a wide range of cultural backgrounds: Germans, Wichí in Argentina, ≠Akhoe Hai||om in Namibia, Quechua in Bolivia, and Kikuyu and Samburu in Kenya. While we have shown how and to what extent children help, their reasons for doing so still need further research. Collecting extensive data on children’s social experiences and combining naturalistic observations with behavioral experiments would be an important step into that direction. While standardized tasks make it possible to compare children’s behavior across different settings, naturalistic observations of actual helping scenarios would provide important insights into the different types of help children provide, and how they grow into their roles as helpers.

Schooling might be an important factor in shaping children’s helping style as well. While attending school reduces the amount of time children spend helping at home, it also creates new opportunities for helping peers—which might often involve providing information. Extending our work to non-school going children to see if they might provide even more instrumental and less informative help than our participants would therefore add another interesting piece to our puzzle. Another important aspect would be to directly compare the way in which children help adults vs. peers. Given the different relationships between children and adults across our settings—from more egalitarian relationships in hunter-gatherer and Western settings, to highly hierarchical ones among pastoralists—and the amount of help children are expected to provide around home, it would be interesting to see if children’s helping behaviors reflect their different cultural norms when interacting with adults.

Linguistic interaction styles might also shape children’s responses (36) and play an important role in the kind of help children provide. Naturalistic observations would help to quantify the extent of verbal exchanges children typically engage in—which might also reflect the observed differences in providing information.

Finally, larger sample sizes would allow us to address questions about individual vs. group level variation (37), which would provide valuable insights into why children help—or fail to do so. The current study found striking similarities in children’s peer helping and matching developmental trends in an experimental task—despite the large diversity of our sample. Across all sites, children reliably helped their partners by providing information or instrumental help and increasingly adapted their behaviors to situational demands. Only providing instrumental help and information at the same time seemed to be a more culture-specific behavior which was more frequent in our urban, industrialized sample as compared to our rural, subsistence-based communities. Our results thus confirm the importance of helping within our uniquely human capacity for collaboration. In addition to honing their collaborative skills, helping also provides children with ample opportunities for social learning—crucial for becoming valued, competent members of their social group. Our work thus provides an important foundation for future research on children’s prosocial development outside the typical settings of the Global North, which is essential for gaining a deeper, wider understanding of human sociality.

## Materials and Methods

### Participants.

Our sample included a total of 336 children, aged 4 to 9 y, from six highly diverse societies in Europe, Africa, and South America (Table 1 and *SI Appendix*).

### Ethics.

The study was conducted in accordance with the Declaration of Helsinki and the ethical guidelines of the German Psychological Society and the Association of German Professional Psychologists. The study did not involve any invasive techniques, ethically problematic procedures, or deception, and therefore did not require approval by an institutional review board in Germany. In all other locations, we obtained permission to conduct our research from the relevant bodies: the National Commission for Science, Technology, and Innovation in Kenya, the Working Group of Indigenous Minorities in Southern Africa in Namibia, and the Ministries of Education in Bolivia and Argentina. After receiving clearance, children were recruited from local pre- and primary schools following local consenting standards (see *SI Appendix* for details).

### Materials.

Materials included four boxes with different opening mechanisms (A1, A2, B1, and B2, see Fig. 2), a marble run, a marble tower, a small container, and five marbles. In addition, there were two plastic cups and two paper bags, one of each with an unobtrusive gap through which marbles could pass. Each marble track consisted of two different boxes (A1 and A2 or B1 and B2), a marble run, a marble tower, a small container, and a plastic cup and a paper bag, one of which was broken (Fig. 1).

### Procedure.

Subjects watched a same-sex, same-aged peer play two rounds of a semistructured game in which players had to extract five marbles from a box, navigate them across a small obstacle course and place them inside another box at the end.

Local research assistants introduced groups of 2 to 3 children to the game and taught them how to open one set of boxes. Half the children (Group A) were introduced to boxes A1 and A2, and the other half of the children (Group B) were introduced to a different set of boxes B1 and B2. During training, children only interacted with their set of boxes and did not encounter any accidents. In the test phase, subjects from one group would watch a peer from the other group interact with the boxes they were familiar with, but which their peers had not encountered before. In addition, there was an invisible cut at the bottom of either the cup or the bag, so that players would encounter two types of accidents: either a closed box which they did not know how to open, or a broken cup/bag through which marbles would spill as they were trying to catch them. In round one, players had to take their marbles out of an open box, place them on a marble run and catch them with a broken cup at its end. Next, they would transfer the marbles to a small bowl, pour them inside the marble tower, and catch them with a paper bag below before placing them inside a closed box at the end of the track. In round two, the players had to take their marbles out of a closed box, place them on a marble run and catch them with a cup at its end. Next, they would transfer the marbles to a small container, pour them inside the marble tower, and catch them with a broken paper bag below before placing them inside an open box at the end of the track.

Subjects were thus presented with two opportunities to help: 1) open the box; and 2) retrieve marbles. Both cases allowed for either instrumental help (opening the box or picking up marbles) or providing information (explaining the opening mechanism or pointing out the location of marbles). While retrieving marbles was a problem which the players could easily solve themselves, they depended on the subject’s support for opening the boxes. We had a total of two trials, each of which included a maximum of two opportunities to help. All trials were videotaped, and verbal interactions were transcribed and translated into English or German by local assistants fluent in the relevant languages.

### Coding.

Situations in which children were struggling to open a closed box or had marbles dropping to the floor were coded as box or marble accidents. Helping events following these accidents were coded as either instrumental helping or informing. In case of a marble accident, instrumental helping would involve children getting up to retrieve any of the scattered marbles. Informing meant that children simply told their partner where some of the marbles had gone or pointed to their location. For box accidents, instrumental helping meant that children took the closed box and tried to open it for their partner, while informing would include any verbal instructions given. Each accident could thus be followed by one of four possible events: a) no help, b) informing only, c) instrumental help only, or d) informing and instrumental help. Helping events were coded as a binary variable (0 = no help provided; 1 = help provided). Additional details can be found in *SI Appendix*.

### Data Analysis.

We used the brms R package (38) to fit a Bayesian categorical regression model to our data. Specifically, we used an outcome variable with four possible values: no helping, informative helping only, instrumental helping only, and both together. The probabilities of the three helping responses relative to the probability of not helping were allowed to vary according to three separate linear functions, each with the type of accident, the participant’s age and sex, and interactions between these as predictor variables. Per-culture random intercepts and random slopes for all noninteraction predictors were included to account for cross-cultural variation. Regularizing priors were used for all parameters (exponential distributions on random effect variances, normal distributions centered on zero on all other terms, full specification in *SI Appendix*). WAIC scores were used to compare the expected out-of-sample predictive accuracy of this model against two reduced forms. One reduced model removed all the predictors but retained per-country random intercepts, while the other retained all the predictor variables of the main model but removed the per-country random effects.

## Supplementary Material

Appendix 01 (PDF)

## Data Availability

Anonymized behavioral data have been deposited in Zenodo (39). All other data are included in the manuscript and/or *SI Appendix*.
