# Supplementary material for: Children from six cultures reliably help their peers—but in different ways
Source: Proc Natl Acad Sci U S A. 2026 Oct 2;123(40):e2515233123. doi: 10.1073/pnas.2515233123 (PMC13643313; doi:10.1073/pnas.2515233123)
Supplement: Supplementary file 1 [file pnas.2515233123.sapp.pdf]

PNAS

Supporting Information for
Children from six cultures reliably help their peers - but in different ways

Henriette Zeidler, Marie Schäfer, Esther Herrmann, Patricia Kanngiesser, Luke Maurits, Daniel Haun, and Michael Tomasello

Daniel Haun
Email: haun@eva.mpg.de

This PDF file includes:

- Supporting text
- Figures S1 to S4
- Tables S1 to S2
- R Script
- SI References

Supporting Information

1 Methods

1.1 Participants

1.2 Study sites/samples

1.2.1 Akhoe Haikom, Namibia

1.2.2 Kikuyu, Kenya

1.2.3 Leipzig, Germany

1.2.4 Quechua, Bolivia

1.2.5 Samburu, Kenya

1.2.6 Wichí, Argentina

1.3 Ethics and consent

1.4 Materials and set-up

1.5 Training and test sessions

1.6 Data recording and coding

1.7 Data analysis

2 References

1 Methods

1.1 Participants

Our sample included a total of 336 children, aged four to nine years, from 6 different societies in Europe, Africa, and South America (see Table S1 and site descriptions below). An additional 8 subjects (Hai||om: 1m, 1f; Kikuyu: 1m, 3f; Samburu: 2m); participated in the test phase but were later dropped from the study because they had switched roles (4 children), due to an experimenter error (2 children), or due to poor video quality (2 children). In each location, 12 additional children (1 male and 1 female per age x 6 age groups) participated in the training, but only served as players during the test.

Since birth certificates were not available for a large part of our sample, we recorded ages in full years (without decimals) based on school records and parents' statements.

1.2 Study sites

1.2.1 ǂAkhoe Hai||om, Namibia (cultural group)

The Hai||om live in the Savannah drylands of Northern Namibia. Traditionally, the Hai||om inhabited the area surrounding the Etosha salt pan. Today, most Hai||om live on small farms or in villages. As many former hunter-gatherer communities, they are politically and socially marginalized and can no longer follow their traditional lifestyle as hunting grounds are owned or occupied by others. Some more rural Hai||om communities have nonetheless maintained many central aspects of their cultural identity. Even though many engage in paid jobs and receive governmental aid (1), foraging is still practiced on a daily basis and both hunting and gathering are still perceived as crucial aspects of Hai||om cultural identity (1, 2). Other cultural elements such as the emphasis on individual autonomy, egalitarianism, and sharing on demand, are practiced regularly (2, 3). Other examples of retained traditional practices are healing trance dances, the traditional kinship and naming system, and a landscape-based system for orientation (2). The language, ǂAkhoe Hai||om, is part of the Khoekhoe cluster of languages. Hai||om children who participated in the present studies lived in a rural settlement with around 300 mostly Hai||om inhabitants. The settlement is located next to a cattle farm which provides seasonal employment for some of the villagers. Children have the opportunity to attend a local school from grade zero to grade five, but local teachers report school attendance to

be low. As is typical for hunter-gatherer societies, children are raised to develop high levels of autonomy and are mostly free to structure their own activities with little emphasis on social obligations or direct instructions from adults (4, 5). Consequently, children spend large amounts of time in multi-age playgroups without adult supervision, often at a distance from their homes. Ball games or other physical activities (hopscotch, rope jumping) are typical favorites.

Children were recruited in the community and tested in a room in the local school (directly adjacent to the settlement) after school hours. A local assistant explained the game in Hai||om. We thank the community on Farm 6 and the Farm 6 school for their support.

1.2.1 Kikuyu, Kenya (cultural group)

The Kikuyu are Kenya's largest ethnic group, living in the central part of the country, and their language (Kikuyu) is part of the Bantu language family. Traditionally, they are small-scale farmers, cultivating maize, beans, and other vegetables and practicing animal husbandry for their subsistence. Although trade and wage work have become increasingly important over the last decades (e.g. paying for building materials, clothes, or school items), agriculture continues to be the main pillar of the Kikuyu economy. Despite many changes over the last years, the family still forms the Kikuyu's basic economic and social unit. The Kikuyu are patrilineal, and fathers are followed by their eldest sons as heads of the family. Typically, male members of the family continue to live in the same village or nearby. Older members of the communities are highly respected (6, 7), but positions in other institutions such as church or government are also causes for respect. Decisions are usually made by the official political bodies, although local councils of elders might be consulted for family or community issues. Although many Kikuyu have moved out of their original homelands, their tribal ties often remain unbroken and their strongest relations are still within their own ethnic group. Although children are highly valued, many families at present decide to restrict the number of children they have due to economic reasons (8). Many children attend nursery school from about four years of age, and almost all of them will go to school once they are five or seven years old. Schooling is free of charge at the primary level (classes 1 to 8). Outside school, children help with various tasks in and around the house or field, take care of their younger siblings, or look after animals - often without adult supervision. At the time of data collection, only few homesteads had electricity, and children spent most of their free time around the family compound, engaging in ball games or role play. Toys were rare, and children used

everyday materials such as buckets, empty cans, small stones, or strings for their games. Children were still expected to be quiet and obedient at home, although parents increasingly tended to value curiosity and self-confidence in school settings (7).

Children were recruited from two primary schools in rural areas near Nanyuki town in Central Kenya and tested in a quiet room at their school after regular school hours. The experimenter used English and Swahili when talking to children, which are the languages of instruction in school. We thank the participating primary schools for their support, and the Kenyan National Committee for Science, Technology, and Innovation (NACOSTI) for approving this research.

1.2.3 Leipzig, Germany (location)

Leipzig is a medium-sized town in the Eastern-Central part of Germany and has a population of more than 550,000 people that has been steadily growing in recent years (9). At the time of the study (in 2015) about 17% of the population had a migration background, but the numbers have been increasing to over 20% in recent years. German is the main language spoken in the city. The economy is based primarily on salaried and wage work with car manufacturing and logistics playing an important role. Leipzig also hosts numerous annual fairs and is known for its book fair. Families are usually nuclear families with one or two children in the family. Parents spend a large part of the day away from home and the majority of children are cared for in nurseries or other child-care settings outside the home from early on. From age six, children attend primary school and spend a lot of time outside the family home. In general, German parents socialize their children to be independent, self-confident, and assertive (10). Playing and learning are viewed as important parts of children's lives, and children are usually not responsible for household tasks until well beyond middle childhood. Most children have their own room, which tends to be filled with toys and games. Outdoor activities typically take place in designated areas (playgrounds, sports fields), and children are hardly ever out of an adult's sight.

Children were recruited through a database of families who had agreed to take part in developmental studies. Children were tested in their local kindergarten. All instructions were in German and children were tested by a native German speaker. We thank all participating kindergartens for their support.

1.2.4 Quechua, Bolivia (cultural group)

The Quechua are the largest of the indigenous groups ("Naciones y Pueblos Indígena Originario Campesinos (NyPIOC)") in Bolivia comprising more than 1.8 million people or ~18% of the population (11). Quechua is an officially recognized language in Bolivia and still actively spoken by more than 1.6 million speakers. The majority of Quechua in Bolivia live in the departments of Cochabamba, Potosí, and Chuquisaca. The main subsistence in rural areas consists of agriculture and in urban centres of wage work. Reciprocity—"ayni" in Quechua—plays an important role in structuring social and economic exchange and represents a shared moral order of how people should interact with each other (12). For example, in the economic realm this includes mutual help with field work. Family structure usually consists of nuclear families, but with parents increasingly migrating to cities or even abroad for better economic prospects, children are increasingly cared for by grandparents or aunts/uncles. Children attend school where they are usually taught in Spanish, but also learn one indigenous Bolivian language (e.g. Quechua) and one foreign language (often English). In rural areas, children often help with subsistence activities and may miss school at certain times of the year because of it (13). Children's free time activities are often interwoven with work, and games - such as hopscotch or ball games - typically involve several children of mixed ages.

The current research took place in villages around Tarata in the department of Cochabamba, at an altitude of approximately 2800 meters. Subsistence in the villages consists primarily of agriculture and, in some cases, pottery.

Children were recruited through local schools and tested in a quiet room in their school. Two experimenters conducted the study and spoke to children in Spanish. We thank the communities and schools in Huayculi and Villa San José, and the district's education secretary for their support.

1.2.5 Samburu, Kenya (cultural group)

The Samburu are a group of Nilotic people predominantly living in the arid lands of Central and Northern Kenya (population size approx. 300.000). Being closely related to the Maasai, their language (Samburu) is a dialect of Maa (belonging to the Nilo-Saharan linguistic phylum). Traditionally Samburu people live as semi-nomadic herders, raising livestock such as cattle, camels and goats that they seasonally move between fertile pastures. Nowadays, the lifestyles of Samburu people are diverse, ranging from a nomadic life with few commodities and based on simple subsistence to the stable, materialistic life

in big cities based on professional jobs. However, Samburu have largely upheld their cultural traditions and, compared to other Kenyan groups, have embraced Westernization to a much lesser degree. Particularly in rural communities, social organization is still characterized by a strict gender- and age-based hierarchy (14). Important resources and decision powers are held by group elders (i.e. male household heads) and many aspects of a person's rights and obligations within communities are determined by social status. The present research was conducted in a rural community near the small town of Wamba in Samburu East. Children's families lived in scattered homesteads about 45-60 minutes walking distance from town. At the time of data collection, compounds did not have electricity; cooking took place on open fire and water had to be carried over considerable distances from nearby water holes or pumps. Many families relied on livestock for subsistence; women sometimes engaged in small-scale gardening around their homes during the rainy season, and some men held paid jobs in local conservancies or engaged in occasional wage work. For the most part, families were sedentary throughout the year, and only some family members (usually young unmarried men) were responsible for moving with the cattle during the dry season. Children were treated leniently until about 3 years of age, but as they grew older, respect for elders and obedience played an important role in their socialization. From around 4 to 6 years of age, girls were expected to help with household task such as fetching water or firewood and looking after younger siblings. Boys typically helped with looking after livestock. From early on, children were free to roam around with their peers, visit other family members, and spent most of their time away from adults. Games usually involved physical activities such as jumping, dancing, or throwing/catching as well as role plays imitating everyday experiences or ceremonies.

All children in the current study attended the local primary school or adjacent preschool in which they were taught in Kiswahili, English and sometimes in their mother tongue Samburu (in preschool). Study sessions were conducted after class and took place in an empty room of the school building. Study instructions were presented to children in Samburu by a local assistant. We thank the local community and primary school in Golgoltim, in particular the teachers and children for their support and participation. Additionally, we thank Richard and Josephine Lesowapir for hosting us during data collection and the Kenyan National Committee for Science, Technology, and Innovation (NACOSTI) for approving this research.

1.2.6 Wichí, Argentina (cultural group)

The Wichí are one of the 35 officially recognized *pueblos originarios* (Indigenous groups) in Argentina and, in contemporary Argentina, live primarily in the provinces of Salta, Formosa, and Chaco. The Wichí language belongs to the Mataguayan language family, was traditionally not a written language and can be considered an endangered language. Traditionally, the Wichí followed a semi-nomadic life-style within fixed territories and subsisted on hunting, gathering, fishing, honey collection and seasonal slash-and-burn horticulture (15). Beginning in the 19th century, the Wichí lost many of their traditional territories due to military actions by the Argentinian government and the increased presence of European settlers. In present day Argentina, there are about 50,000 Wichí (16). Most Wichí are now sedentary and live in urban dwellings or in often impoverished communities throughout the Chaco. Families subsist on selling traditional crafts, gathering, some wage labour, and governmental assistance for families (asignación familiar). Deforestation and large-scale soy plantations in the Chaco region are a threat to Wichí livelihoods and health (e.g., due to pesticides). The present study was conducted in Misión Chaqueña, which is located in the Gran Chaco region in Salta Province, Northern Argentina. Misión Chaqueña was founded as the first Anglican mission in the Chaco in 1914 and today more than 1500 Wichí live in the community. The majority of the inhabitants are ethnically Wichí. Residential groups are based on kin-relations between female family members (17) and usually occupy a parcel of land with small houses (1-2 rooms) made of mudbricks, bricks, or metal sheets. Most houses have electricity, but often no running water. The community relies on various wells for their water supply, but often wells are not deep enough to provide an uncontaminated water supply and water is often scarce in the hot summer months. The community has a kindergarten, primary and secondary school. School is mandatory from age six. Children speak Wichí at home. Once children enter kindergarten (from age 4) or school (from age 6), they are taught in Spanish. Most classes have bi-lingual teaching assistants in addition to Spanish-speaking teachers. Though all children learn to read and write in Spanish, but proficiency can vary. The majority of children's daily activities besides school are outdoors (18). From around school age, children move around the community by themselves or in peer groups - typically without adult supervision. Wichí children are not explicitly taught, but learn by participating in daily activities (19). Most children are familiar with computers (e.g., there is a computer room and computer lessons at school) and mobile phones.

Children were tested in a room in their local school. Instructions were presented by two local experimenters who used Spanish when talking to children. We thank the Comunidad Misión Chaqueña, the school No. 4528, and the Ministerio de Educación, Cultura, Ciencia y Tecnología of Salta province for their support.

Table S1. *Overview of study participants*

**Population**	**Location**	**Social organization**	**Economic base**	**Number of children per family**	**School enrolment (county level)**	**N (female)**	**M Age (SD)**
≠Akhoe Hai||om	Namibia, Africa	village	hunter-gatherers, some wage work	4.1	67%	56 (28)	6.6 (1.53)
Germans	Germany, Europe	town	wage work	1.5	100%	60 (31)	6.2 (1.68)
Kikuyu	Kenya, Africa	village	agriculture, wage work	2.7	94%	58 (29)	6.4 (1.73)
Samburu	Kenya, Africa	village	herding, some wage work	6.3	57%	68 (30)	6.5 (1.65)
Quechua	Bolivia, South America	village	agriculture, wage work	3.5	83%	47 (24)	6.4 (1.65)
Wichí	Argentina, South America	village	craft production, some wage work	4.5	87%	47 (23)	6.2 (1.61)
**ALL**						**336 (165)**	**6.4**

1.3 Ethics & consent

Our study was conducted in accordance with the Declaration of Helsinki and the ethical guidelines of the German Psychological Society and the Association of German Professional Psychologists. Since the methods did not involve any invasive techniques, ethically problematic procedures, or deception, the study did not require approval by an institutional review board in Germany. In all other locations, we obtained permission to conduct the research from the relevant bodies: the National Commission for Science, Technology, and Innovation (NACOSTI) in Kenya, the Working Group of Indigenous Minorities in Southern Africa (*WIMSA*) in Namibia, and the Ministries of Education in Bolivia and Argentina. Participants were recruited through local pre- and primary schools (≠Akhoe Hai||om, Kikuyu, Quechua, Samburu, Wichí) and through a database of parents who had agreed to take part in developmental studies (Leipzig). In Leipzig, at least one legal guardian gave informed consent prior to their child taking part in the study. In ≠Akhoe Hai||om, Kikuyu, Quechua, Samburu, and Wichí communities, we obtained permission to conduct our research from education or science ministries, local school boards, and/or community representatives. Informed consent for children's study participation was given by the schools' head-teachers. Children received small gifts (sweets or stickers) as a thank you for their participation. Participating schools received a small donation (e.g., books) as a thank you for their help. Local communities usually received a small donation as a thank you for hosting the research team.

1.4 Materials and set-up

Materials included four boxes with different opening mechanisms (A1, A2, B1, and B2), a marble run, a marble tower, a small container, and five marbles. In addition, there were two plastic cups and two paper bags, one of each with an unobtrusive gap through with marbles could pass. Both rounds of the game were played with the same basic setup (see Figures S1 and S3).

The boxes (A 1 and A2 or B1 and B2) marked the very beginning and end of the track. The marble run and the marble tower were placed around 2 meters apart on low tables in the middle of the track, with either a cup or a bag below for catching the marbles. An additional small bowl was placed between the two tables, so that children would always have an intact vessel for collecting marbles and transferring them to the tower.

The only difference between rounds 1 and 2 was that accidents were staged at different points of the track: in round 1, the cup next to the marble run was broken, and the box at the end was closed; and in round 2, the box at the beginning was closed and the paper bag next to the marble tower was broken (see Figures S2 and S4).

1.5 Training and test sessions

The study consisted of two sessions: a training session (day 1) and a test session (day 2), with a minimum of 1 day between them. At each site, the study was conducted by a main experimenter (E). In some cases, a second experimenter supported E by getting children, combining them to dyads, or helping with translations (see study site descriptions, section 1.2, for details).

Training session

Children in the right age categories were randomly divided into two equally large groups (A and B). Local research assistants then introduced smaller subgroups of 2 to 3 children to the game and taught them how to open one set of boxes. Half the children (Group A) were introduced to boxes A1 and A2, and the other half of the children (Group B) were introduced to a different set of boxes B1 and B2. During training, children only interacted with their set of boxes, and did not encounter any accidents.

Test session

During the actual test, subjects from one group would watch a same-aged peer from the other group interact with the boxes they were familiar with, but which their peer

had not encountered before. Subjects from group A thus watched a peer from group B trying to open boxes A1 and A2, and subjects from group B watched a peer from group A struggle with boxes B1 and B2. In addition, there was an invisible cut at the bottom of either the cup or the bag, so that players would encounter two types of accidents: either a *closed box* which they did not know how to open, or a *broken cup/bag* through which marbles would spill as they were trying to catch them. In round one, players had to take their marbles out of an open box, place them on a marble run and catch them with a *broken cup* at its end. Next, they would transfer the marbles to a small bowl, pour them inside the marble tower, and catch them with a paper bag below before placing them inside a *closed box* at the end of the track. In round two, the players had to take their marbles out of a *closed box*, place them on a marble run and catch them with a cup at its end. Next, they would transfer the marbles to a small container, pour them inside the marble tower, and catch them with a *broken paper bag* below before placing them inside an open box at the end of the track.

Subjects were thus presented with two opportunities to help: 1) open the box; and 2) retrieve marbles. Both cases allowed for either instrumental help (opening the box or picking up marbles) or providing information (explaining the opening mechanism or pointing out the location of marbles). While retrieving marbles was a problem which the players could easily solve themselves, they depended on the subject's support for opening the boxes. We had a total of two trials, each of which included a maximum of two opportunities to help.

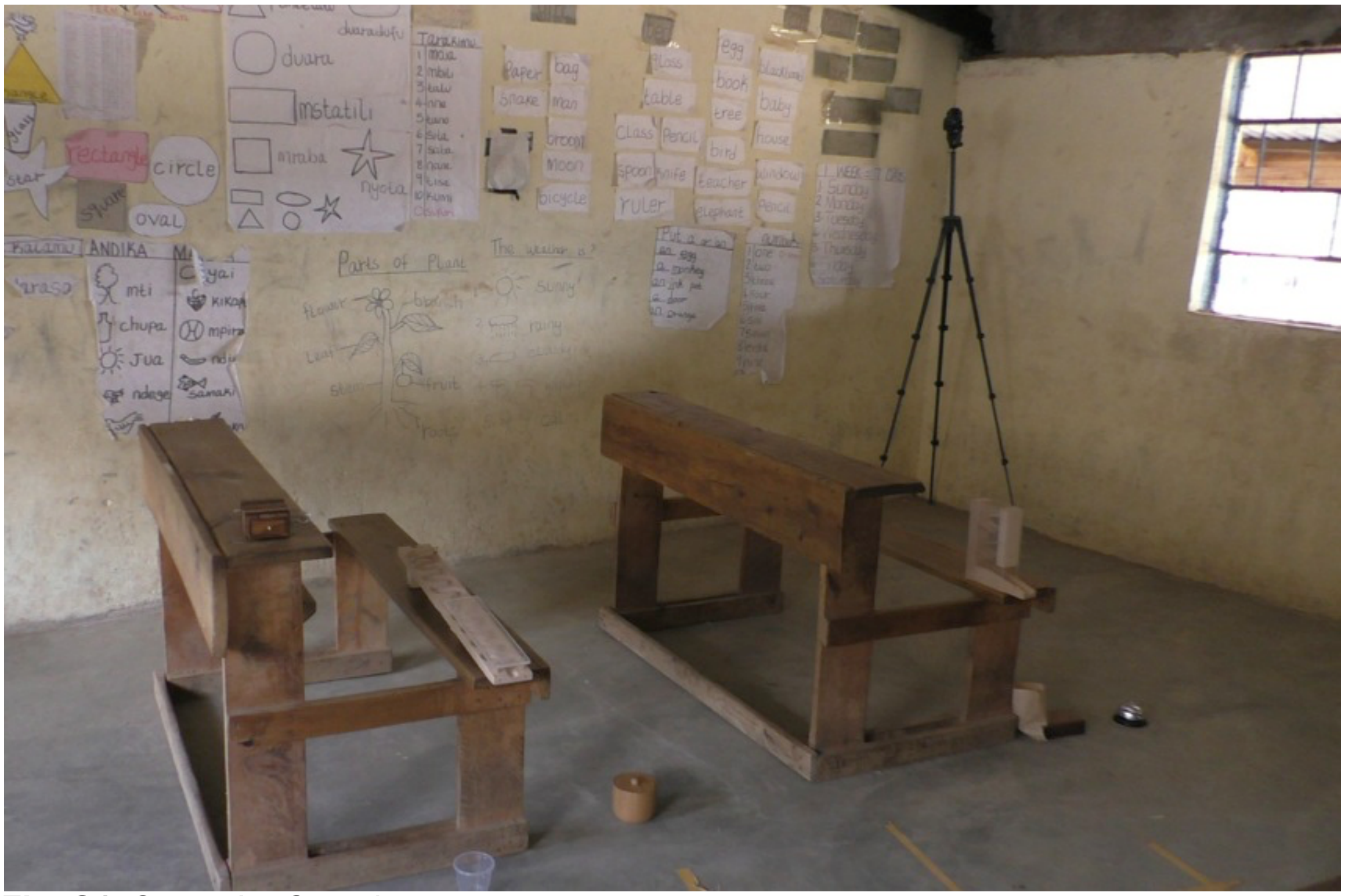

Fig. S1. Setup A - Overview

	box A1	marble run	bowl	marble tower	box A2
	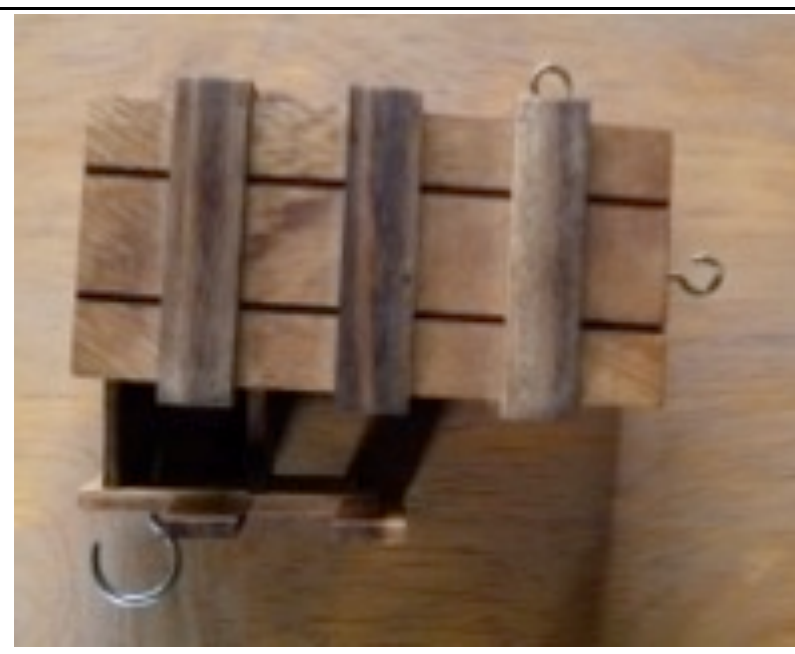	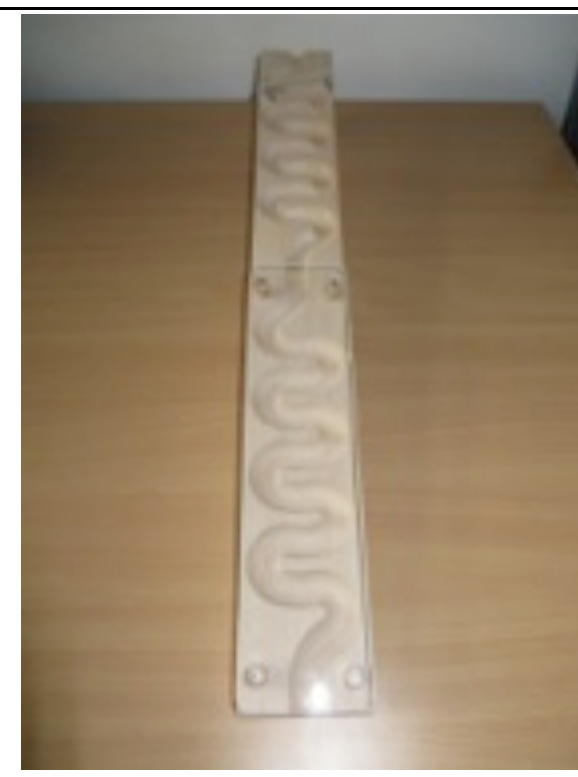	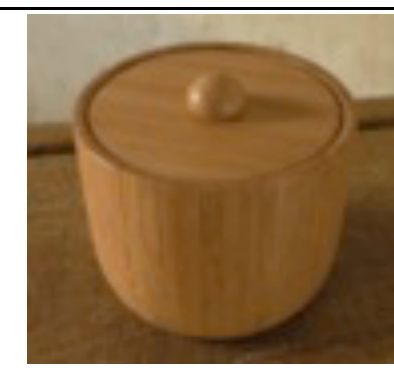	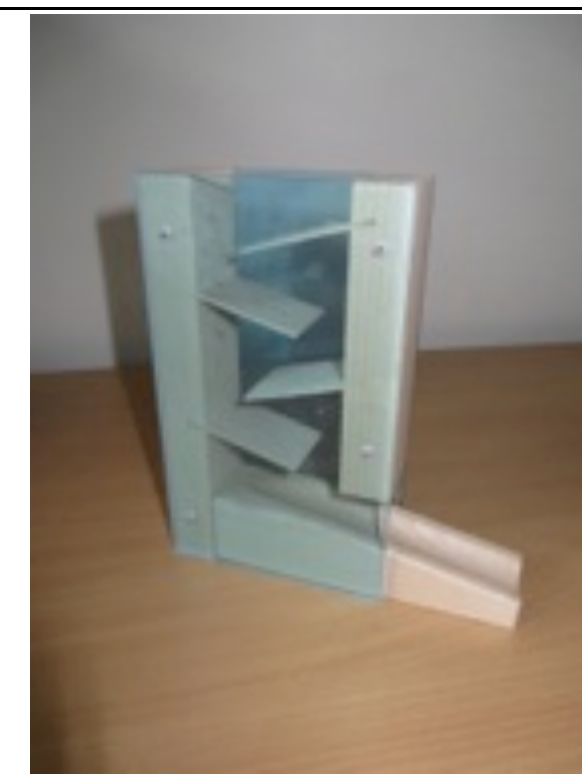	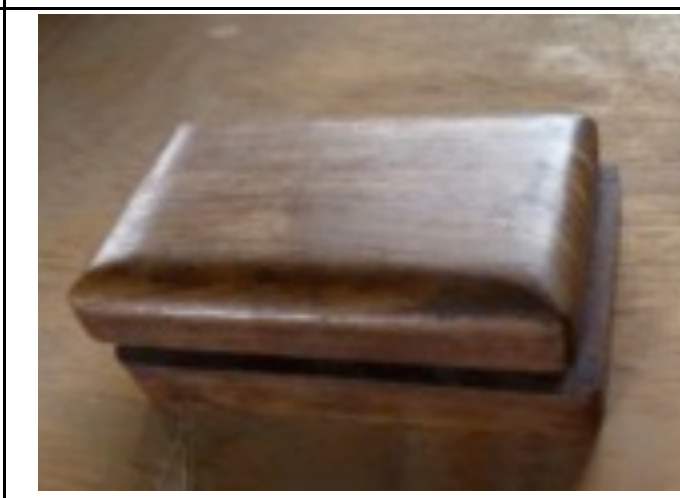
		**cup (broken)**		**bag (broken)**	
		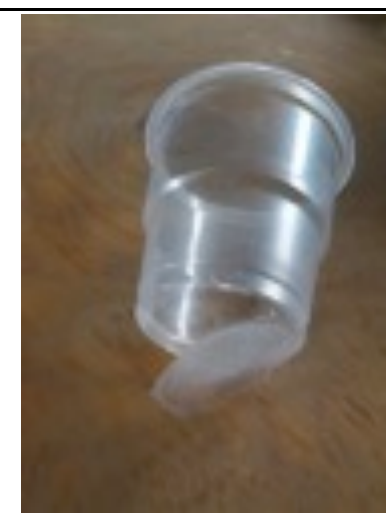		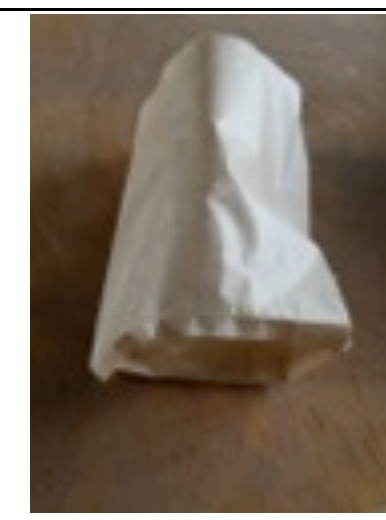	
Round 1	box closed, opening mechanism unknown	cup ok		bag broken, marbles spill on floor	box open
Round 2	box open	cup broken, marbles spill on floor		bag ok	box closed, opening mechanism unknown

Fig. S2. Setup A - Details

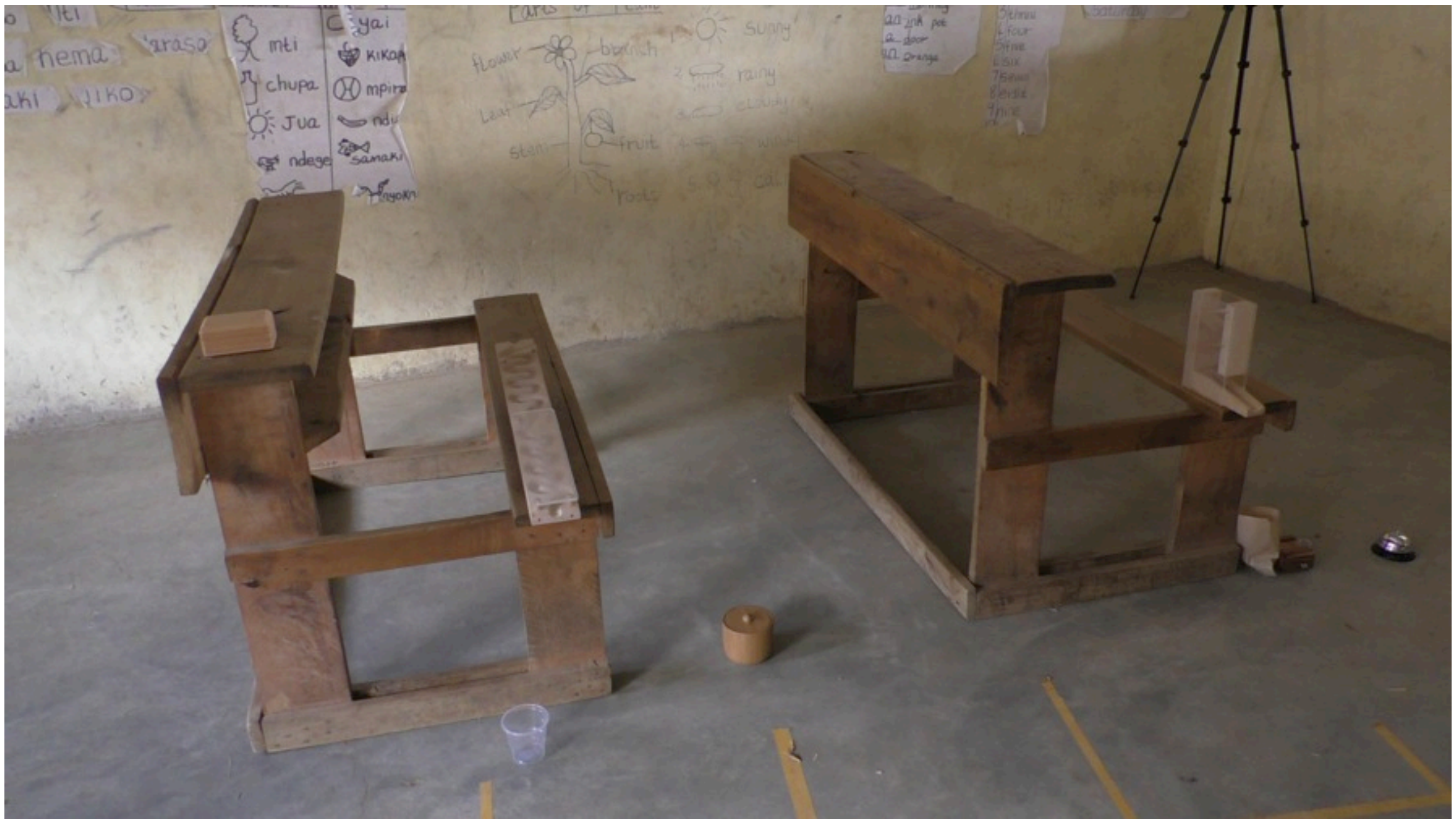

Fig. S3. Setup B - Overview

	box B1	marble run	bowl	marble tower	box B2
	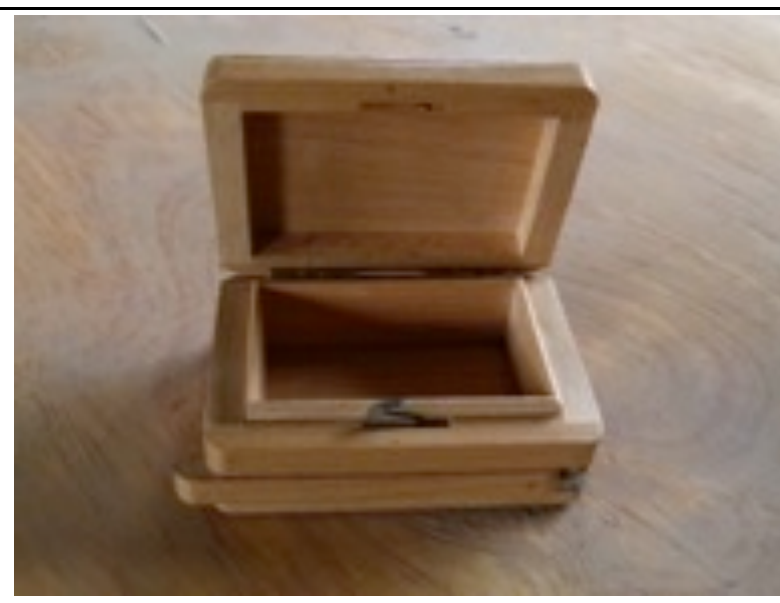	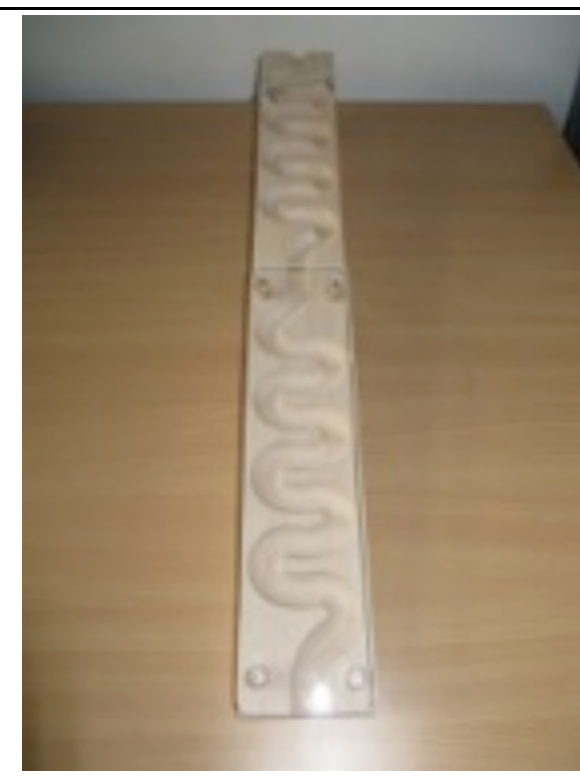	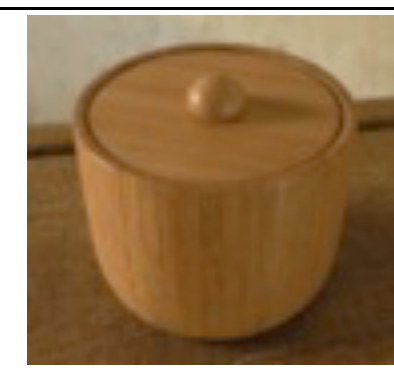	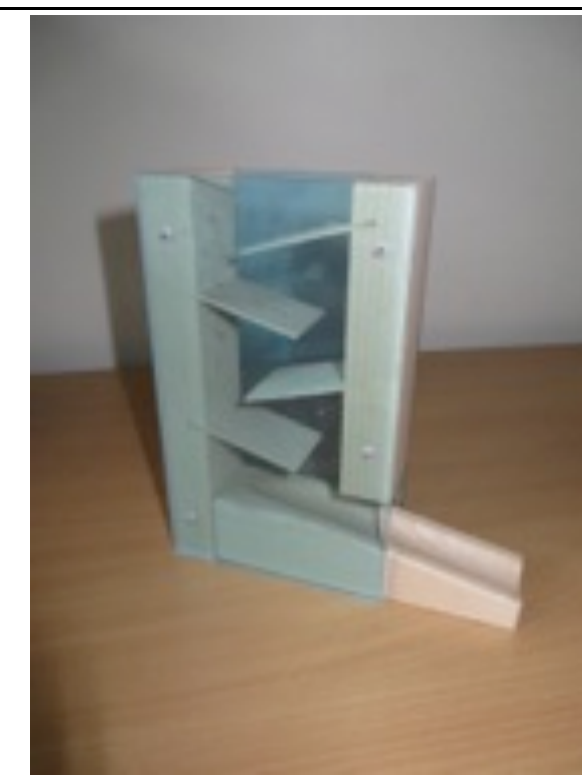	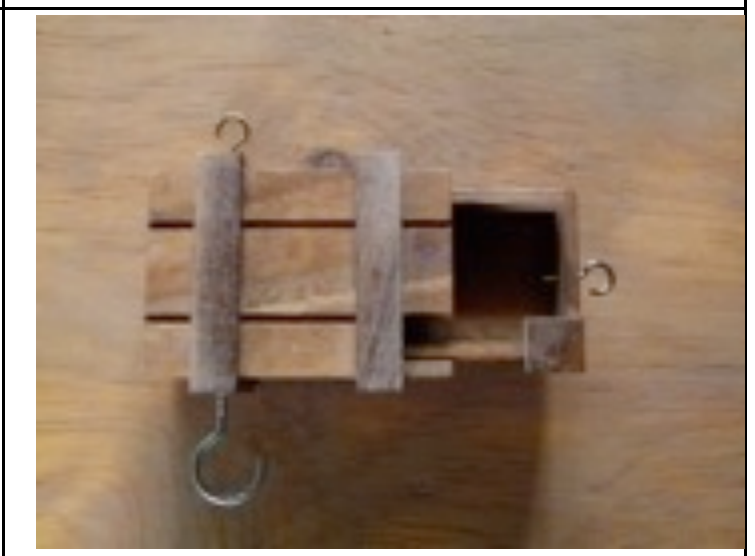
		**cup (broken)**		**bag (broken)**	
		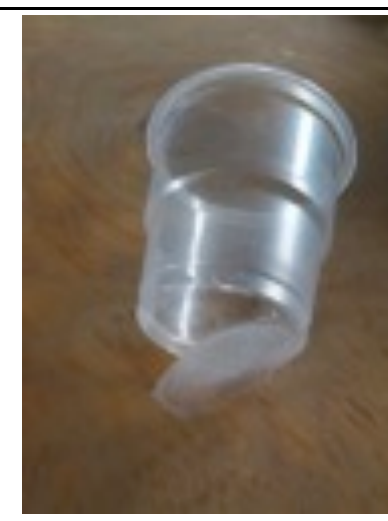		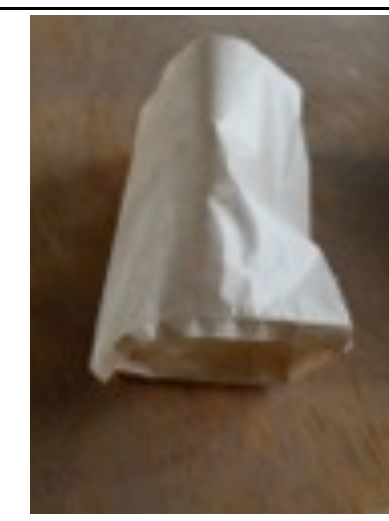	
Round 1	box open	cup broken, marbles spill on floor		bag ok	box closed, opening mechanism unknown
Round 2	box closed, opening mechanism unknown	cup ok		bag broken, marbles spill on floor	box open

Fig. S4. Setup B - Details

1.6 Data recording and coding

All trials were recorded with two video cameras: one overview camera, and one focusing on the helper. Native speakers of the relevant local languages later transcribed and translated the children's utterances. Utterances by Wichí and Quechua children were translated to Spanish and from Spanish to German. Utterances from Samburu, Kikuyu and Hai||om children were translated to English. Utterances by German children were transcribed only. Behaviors were coded in Excel according to the coding scheme provided in Table S2. Inter-rater agreement (Cohen's Kappa) was high or very high across categories (20): Marble accidents: $\kappa = 1.000$; Box accidents: $\kappa \approx 0.98$; Informative help marbles: $\kappa \approx 0.66$; Instrumental help marbles: $\kappa \approx 0.94$; Informative help box: $\kappa \approx 0.91$; Instrumental help box: $\kappa \approx 0.99$.

Table S2. *Coding scheme*

**Code**	**Description**
Marble accident	- cup / bag breaks, marbles spill on floor
Box accident	- player twists and turns box several times
Informative help marbles	- observer tells player where marbles have gone<br>- observer points to location of marbles
Instrumental help marbles	- observer gets up and picks up marble(s)
Informative help box	- observer advises player on how to open box
Instrumental help box	- observer touches/takes box and opens it (or at least tries to open it)

1.7 Data analysis

We used the brms R package (21) to fit a Bayesian categorical regression model to our data. Specifically, we used an outcome variable with four possible values: no helping, informative helping only, instrumental helping only, and both together. The probabilities of the three helping responses relative to the probability of not helping were allowed to vary according to three separate linear functions, each with the type of accident, the participant's age and sex, and interactions between these as predictor variables. Per-culture random intercepts and random slopes for all non-interaction predictors were included to account for cross-cultural variation. Regularizing priors were used for all parameters (exponential distributions on random effect variances, normal distributions centered on zero on all other terms, full specification in SI). Widely Applicable Information Criterion (WAIC) scores were used to compare the expected out-of-sample predictive accuracy of this model against two reduced forms. One reduced model removed all the predictors but retained per-country random intercepts, while the other retained all the predictor variables of the main model but removed the per-country random effects.

Cross Cultural Helping Study Analysis

Luke Maurits

2026-09-17

Model description and comparisons

We used the `brms` R package to fit a Bayesian categorical regression model to our data. Specifically, we used an outcome variable with four possible values: no helping, informational helping only, instrumental helping only, and both together. The probabilities of the three helping responses relative to the probability of not helping were allowed to vary according to three linear models with the type of accident, the participant's age and sex, and interactions between these as predictor variables. Per-country random intercepts and random slopes for all non-interaction predictors were included to account for cross-cultural variation.

Widely Applicable Information Criterion (WAIC) scores were used to compare the expected out-of-sample predictive accuracy of this model against two reduced forms. One reduced model removed all the predictors but retained per-country random intercepts, while the other retained all the predictor variables of the main model but removed the per-country random effects. The main model's predictive performance significantly exceeded the other two models, suggesting that both cross-cultural variation in behaviour and systematic effects of our predictor variables are necessary to account for the data:

```
kable(loo_compare(m, m_monocult, m_intercept, criterion="waic"), format.args=list(digits=4))
```

model	elpd_diff	se_diff	p_worse	diag_diff	diag_elpd	elpd_waic	se_elpd_waic	p_waic	se_p_waic	waic	se_waic
m	0.00	0.000	NA			-1145	25.72	43.94	2.084	2291	51.45
m_monocult	-46.26	9.666	1			-1192	24.22	21.77	1.205	2383	48.44
m_intercept	-72.84	13.591	1			-1218	23.90	12.24	0.509	2437	47.81

The full set of model parameter intercepts is presented at the end of this document, but for ease of interpretation we focus primarily on the model's predicted probabilities of different helping responses for different accidents and demographics. We begin by focussing on the probability of participants rendering *any* kind of help, which is simply the sum of the probabilities of the final three of the model's four possible outcomes.

Model predictions of helping

Predictions at mean age

Before considering developmental trends in helping behaviour, we first provide an overview of helping behaviour by focusing on the model's predictions for children aged 6.5 years (the mid point of the sampled age interval of 4 to 9 years), marginalising over sex differences:

```
nd <- expand_grid(z.age=0, z.sex=c(-0.5, 0.5), z.accident_type=c(-0.5, 0.5), culture=unique(d2$culture))
avg_predictions(m, newdata=nd, by=c("culture", "z.accident_type")) %>%
    get_draws() %>%
    filter(group == 1) %>%
    mutate(draw = 1 - draw) %>%
    group_by(culture, z.accident_type) %>%
```

```
    summarise(help_any = mean(draw),
          help_any_min = quantile(draw, p=0.025),
          help_any_max = quantile(draw, p=0.975),
          help_any_dir = mean(draw > 0.5)) %>%
    mutate(sumstr = sprintf("%.2f (%.2f - %.2f, %.2f)", help_any, help_any_min, help_any_max, help_any_d
           accident_type = if_else(z.accident_type == -0.5, "marbles", "box")) %>%
    pivot_wider(id_cols = "culture", names_from = "accident_type", values_from= "sumstr") %>%
    arrange(culture) %>%
    kable(caption="Estimated probabilities of displaying any kind of helping across cultures and acciden
```

```
## `summarise()` has regrouped the output.
## i Summaries were computed grouped by culture and z.accident_type.
## i Output is grouped by culture.
## i Use `summarise(.groups = "drop_last")` to silence this message.
## i Use `summarise(.by = c(culture, z.accident_type))` for per-operation grouping (`?dplyr::dplyr_by`)
```

Table 2: Estimated probabilities of displaying any kind of helping across cultures and accident types for children of mean age (6.5 years) (posterior mean and 95% HPD interval)

culture	marbles	box
German	0.62 (0.53 - 0.70, 1.00)	0.85 (0.80 - 0.90, 1.00)
Haikom	0.70 (0.62 - 0.78, 1.00)	0.84 (0.79 - 0.89, 1.00)
Kikuyu	0.45 (0.36 - 0.53, 0.09)	0.74 (0.68 - 0.80, 1.00)
Quechua	0.76 (0.69 - 0.83, 1.00)	0.88 (0.83 - 0.92, 1.00)
Samburu	0.61 (0.53 - 0.68, 1.00)	0.81 (0.76 - 0.86, 1.00)
Wichi	0.67 (0.58 - 0.75, 1.00)	0.84 (0.78 - 0.89, 1.00)

In Table 2 we see that 6.5 year old children were, generally speaking, helpful: the 95% HPD intervals for the probability of offering help (either informative, instrumental, or both) are entirely above chance for both kinds of accident across every culture, with the single exception of Kikuyu children reacting to marble accidents, where the interval includes, and is mostly below, chance. Children from every culture were more likely to help in response to box accidents than to marble accidents: the 95% HPD intervals for the effect of accident type on all three forms of helping excluded zero.

Predictions marginalising out age

As an alternative to focusing on predictions at the "central" age of 6.5 years (which is only "central" with respect to our arbitrary sampling window), we can also consider posterior predictions when marginalising over development:

```
nd <- expand_grid(age=seq(4, 9, 0.5), z.sex=c(-0.5, 0.5), z.accident_type=c(-0.5, 0.5), culture=unique(d
nd$z.age <- (nd$age - 6.5)/2.5
avg_predictions(m, newdata=nd, by=c("culture", "z.accident_type")) %>%
    get_draws() %>%
    filter(group == 1) %>%
    mutate(draw = 1 - draw) %>%
    group_by(culture, z.accident_type) %>%
    summarise(help_any = mean(draw),
          help_any_min = quantile(draw, p=0.025),
          help_any_max = quantile(draw, p=0.975),
          help_any_dir = mean(draw > 0.5)) %>%
    mutate(sumstr = sprintf("%.2f (%.2f - %.2f, %.2f)", help_any, help_any_min, help_any_max, help_any_d
```

```
          accident_type = if_else(z.accident_type == -0.5, "marbles", "box")) %>%
    pivot_wider(id_cols = "culture", names_from = "accident_type", values_from= "sumstr") %>%
    arrange(culture) %>%
    kable(caption="Estimated probabilities of displaying any kind of helping across cultures and acciden
```

```
## `summarise()` has regrouped the output.
## i Summaries were computed grouped by culture and z.accident_type.
## i Output is grouped by culture.
## i Use `summarise(.groups = "drop_last")` to silence this message.
## i Use `summarise(.by = c(culture, z.accident_type))` for per-operation grouping (`?dplyr::dplyr_by`)
```

Table 3: Estimated probabilities of displaying any kind of helping across cultures and accident types, marginalising out develomental effects (posterior mean, 95% HPD interval and posterior probability that helping probability is above 0.5)

culture	marbles	box
German	0.62 (0.54 - 0.69, 1.00)	0.85 (0.80 - 0.90, 1.00)
Haikom	0.70 (0.62 - 0.78, 1.00)	0.84 (0.79 - 0.89, 1.00)
Kikuyu	0.45 (0.38 - 0.53, 0.12)	0.75 (0.68 - 0.81, 1.00)
Quechua	0.76 (0.68 - 0.82, 1.00)	0.88 (0.84 - 0.92, 1.00)
Samburu	0.61 (0.53 - 0.67, 1.00)	0.81 (0.76 - 0.86, 1.00)
Wichi	0.67 (0.59 - 0.75, 1.00)	0.84 (0.79 - 0.89, 1.00)

In Table 3 we see that children within with the 4-9 year old age window were, generally speaking, helpful. The box accident results are essentially identical to those in Table 2, with 95% HPD intervals for the probability of offering any kind of help being above 0.5 across every culture. The marble accident results are somewhat different (the reason for which is obvious in light of the developmental trends discussed below), with wider 95% HPD intervals. However, there is still modest to strong evidence in favour of overall helpfulness, with the posterior probability that helping rates exceed 0.5 ranging betwen 0.84 and 1.00 for all cultures except Kikuyu. There we in fact have weak evidence that helping rates are below 0.5 (posterior probability 0.7).

Developmental trends

Finally we consider developmental effects, first by visualisation:

```
nd <- expand_grid(z.age=seq(-1, 1, 0.1), z.sex=c(-0.5, 0.5), z.accident_type=c(-0.5, 0.5), culture=uniq
int_dev_preds <- avg_predictions(m, newdata=nd, by=c("culture", "z.accident_type", "z.sex", "z.age")) %
    as_tibble() %>%
    tidy_labels() %>%
    filter(group == 1) %>%
    mutate(help_any = 1 - estimate)
ggplot(int_dev_preds) +
    geom_line(aes(x=age, y=help_any, colour=culture, linetype=sex)) +
    facet_wrap(~accident_type) +
    labs(caption="Developmental trends in probability of helping in any way.")
```

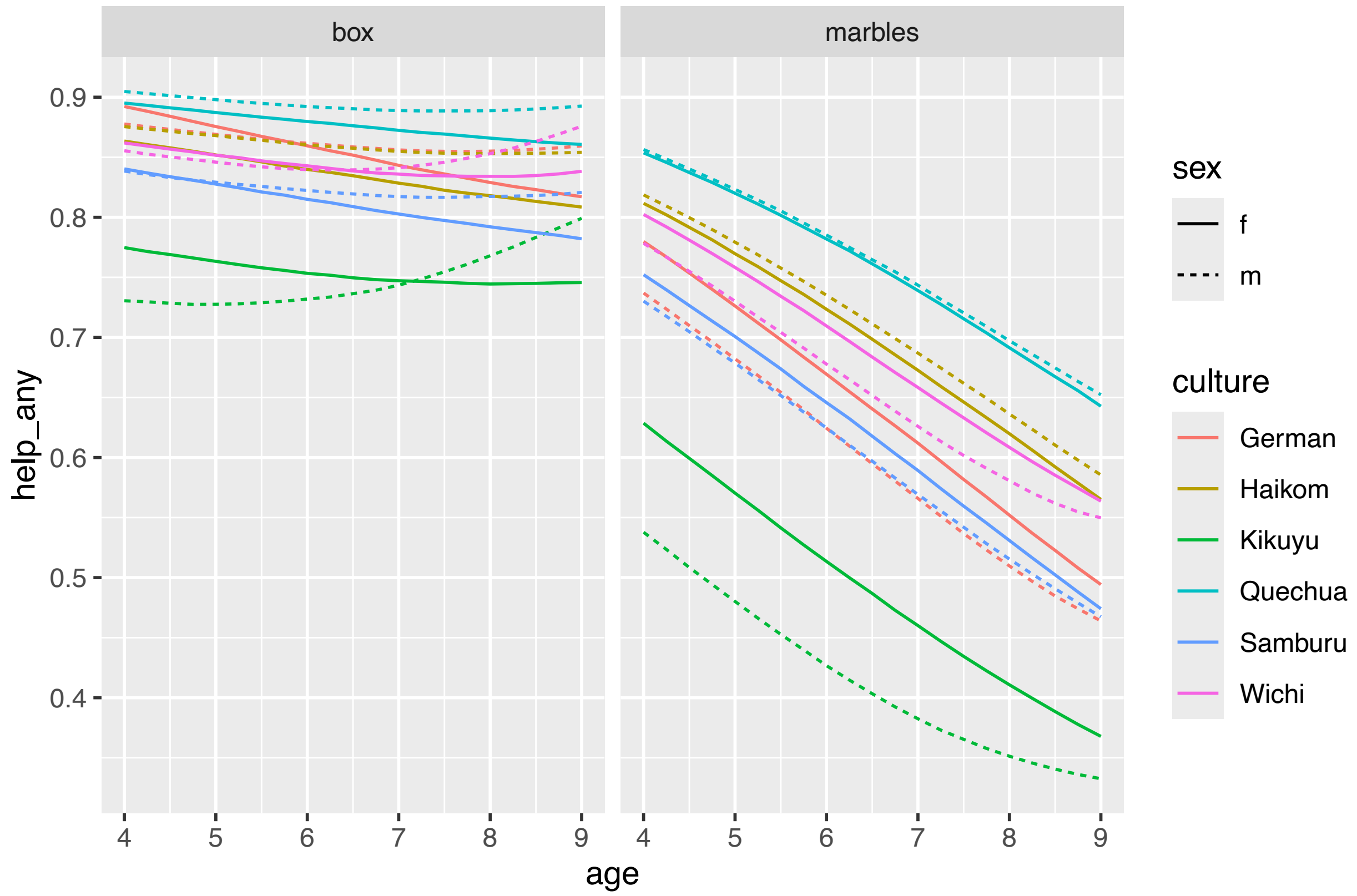

Developmental trends in probability of helping in any way.

The probability of helping in response to box accidents remained relatively steady across the full 4 to 9 year old age range in all cultures. In contrast, helping in response to marble accidents decreased substantially across the age range in all cultures. Table 4 below shows the posterior mean and 95% HPD estimates for the decrease in helping probability between the ages of 4 and 9 for all cultures. Every HPD interval excludes zero, typically by 0.2 or more. The extent of the decrease ranged between 0.20 and 0.28.

```
nd <- expand_grid(z.age=c(-1, 1), z.sex=c(-0.5, 0.5), z.accident_type=c(-0.5, 0.5),
          culture=unique(d2$culture)) %>%
    mutate(accident_type = if_else(z.accident_type == -0.5, "marbles", "box"))
contrast_preds <- avg_comparisons(m, newdata=nd, variable=list(z.age=c(1, -1)),
                    by=c("culture", "accident_type")) %>%
    get_draws() %>%
    filter(group == 1) %>%
    group_by(culture, accident_type) %>%
    summarise(help_any_contrast = mean(draw),
          help_any_contrast_min = quantile(draw, p=0.025),
          help_any_contrast_max = quantile(draw, p=0.975),
          help_any_contrast_dircert = mean(sign(draw) == sign(mean(draw))))
```

```
## `summarise()` has regrouped the output.
## i Summaries were computed grouped by culture and accident_type.
## i Output is grouped by culture.
## i Use `summarise(.groups = "drop_last")` to silence this message.
## i Use `summarise(.by = c(culture, accident_type))` for per-operation grouping (`?dplyr::dplyr_by`) i
```

```
box_table <- contrast_preds %>%
    filter(accident_type == "box") %>%
```

```
    mutate(`Box decrease` = sprintf("%.2f (%.2f - %.2f, %.2f)", help_any_contrast, help_any_contrast_mi
    select(culture, `Box decrease`)
marble_table <- contrast_preds %>%
    filter(accident_type == "marbles") %>%
    mutate(`Marble decrease` = sprintf("%.2f (%.2f - %.2f, %.2f)", help_any_contrast, help_any_contrast_
    select(culture, `Marble decrease`)
left_join(box_table, marble_table) %>%
    arrange(culture) %>%
    kable(caption="Estimated decreases in marble accident helping probabilities between the ages of 4 a
```

```
## Joining with `by = join_by(culture)`
```

Table 4: Estimated decreases in marble accident helping probabilities between the ages of 4 and 9 (posterior mean and 95% HPD interval)

culture	Box decrease	Marble decrease
German	0.05 (-0.05 - 0.15, 0.83)	0.27 (0.14 - 0.41, 1.00)
Haikom	0.04 (-0.07 - 0.14, 0.78)	0.23 (0.10 - 0.36, 1.00)
Kikuyu	-0.02 (-0.16 - 0.11, 0.61)	0.23 (0.07 - 0.37, 1.00)
Quechua	0.02 (-0.06 - 0.10, 0.71)	0.20 (0.08 - 0.32, 1.00)
Samburu	0.04 (-0.07 - 0.15, 0.75)	0.27 (0.13 - 0.40, 1.00)
Wichi	0.00 (-0.10 - 0.11, 0.51)	0.23 (0.09 - 0.37, 1.00)

Separating out the three kinds of helping

We know consider separately probabilities for each of the three possible helping responses.

Cultural variation

Our data shows strong evidence of cross-cultural variation in the forms of helping behaviour, with the 95% HPD intervals for several culture's random intercepts excluding zero. In particular, Kikyu children were less likely to to offer instrumental help than the population mean, while Quechua children were more likely to offer it. German children were more likely to offer informative and instrumental help together than the population mean.

Box at mean age

As shown in Table 5 below, instrumental helping was the most probable kind of helping offered by children of mean age in response to box accidents, and was particularly frequently offered by Quechua children (posterior mean probability 0.72). Nevertheless, the other responses, while rarer, were not exceptionally unlikely. German children offered both informational and instrumental help together at more than 3 times the rate of children from any other culture. Kikuyu children, while being the least likely to offer help of any kind for box accidents (see Table 2), were the most likely to offer informational help alone.

```
nd <- expand_grid(z.age=0, z.sex=c(-0.5, 0.5), z.accident_type=c(-0.5, 0.5), culture=unique(d2$culture)
mean_age_preds <- avg_predictions(m, newdata=nd, by=c("culture", "z.accident_type")) %>%
    as_tibble() %>%
    filter(group > 1) %>%
    mutate(helping_type = case_when(group == 2 ~ "info",
                    group == 3 ~ "inst",
                    group == 4 ~ "both"),
           sumstr = sprintf("%.2f (%.2f - %.2f)", estimate, conf.low, conf.high),
```

```
          accident_type = if_else(z.accident_type == -0.5, "marbles", "box"))
mean_age_preds %>%
    filter(accident_type == "box") %>%
    pivot_wider(id_cols = "culture", names_from = "helping_type", values_from= "sumstr") %>%
    arrange(culture) %>%
    kable(caption="Estimated probabilities of different helping responses to box-type accidents across c
```

Table 5: Estimated probabilities of different helping responses to box-type accidents across cultures (posterior mean and 95% HPD interval)

culture	info	inst	both
German	0.09 (0.06 - 0.14)	0.42 (0.34 - 0.50)	0.34 (0.25 - 0.43)
Haikom	0.10 (0.06 - 0.14)	0.64 (0.55 - 0.72)	0.10 (0.06 - 0.17)
Kikuyu	0.17 (0.12 - 0.23)	0.44 (0.35 - 0.54)	0.13 (0.07 - 0.20)
Quechua	0.08 (0.05 - 0.12)	0.71 (0.63 - 0.79)	0.09 (0.05 - 0.15)
Samburu	0.12 (0.08 - 0.16)	0.58 (0.51 - 0.65)	0.11 (0.07 - 0.17)
Wichi	0.11 (0.07 - 0.17)	0.65 (0.56 - 0.73)	0.07 (0.03 - 0.13)

Box maginalising out development

```
nd <- expand_grid(age=seq(4, 9, 0.5), z.sex=c(-0.5, 0.5), z.accident_type=c(-0.5, 0.5), culture=unique(c
nd$z.age <- (nd$age - 6.5)/2.5
margdev_preds <- avg_predictions(m, newdata=nd, by=c("culture", "z.accident_type")) %>%
    as_tibble() %>%
    filter(group > 1) %>%
    mutate(helping_type = case_when(group == 2 ~ "info",
                    group == 3 ~ "inst",
                    group == 4 ~ "both"),
          sumstr = sprintf("%.2f (%.2f - %.2f)", estimate, conf.low, conf.high),
          accident_type = if_else(z.accident_type == -0.5, "marbles", "box"))
margdev_preds %>%
    filter(accident_type == "box") %>%
    pivot_wider(id_cols = "culture", names_from = "helping_type", values_from= "sumstr") %>%
    arrange(culture) %>%
    kable()
```

culture	info	inst	both
German	0.11 (0.07 - 0.16)	0.41 (0.33 - 0.49)	0.34 (0.25 - 0.43)
Haikom	0.11 (0.07 - 0.15)	0.63 (0.54 - 0.71)	0.10 (0.06 - 0.17)
Kikuyu	0.19 (0.14 - 0.25)	0.43 (0.35 - 0.53)	0.12 (0.07 - 0.19)
Quechua	0.09 (0.06 - 0.13)	0.70 (0.62 - 0.78)	0.09 (0.05 - 0.15)
Samburu	0.12 (0.09 - 0.17)	0.58 (0.50 - 0.65)	0.11 (0.07 - 0.17)
Wichi	0.15 (0.10 - 0.21)	0.63 (0.54 - 0.71)	0.07 (0.03 - 0.12)

Marbles at mean age

As shown in Table 6 below, children's response to marble accidents was much less varied. Instrumental helping alone was by far the most frequent response across all cultures.

```r
mean_age_preds %>%
    filter(accident_type == "marbles") %>%
    pivot_wider(id_cols = "culture", names_from = "helping_type", values_from= "sumstr") %>%
    arrange(culture) %>%
    kable(caption="Estimated probabilities of different helping responses to marble-type accidents acro
```

Table 7: Estimated probabilities of different helping responses to marble-type accidents across cultures (posterior mean and 95% HPD interval)

culture	info	inst	both
German	0.04 (0.02 - 0.07)	0.54 (0.45 - 0.62)	0.04 (0.01 - 0.08)
Haikom	0.03 (0.02 - 0.06)	0.65 (0.56 - 0.74)	0.02 (0.01 - 0.04)
Kikuyu	0.06 (0.04 - 0.10)	0.36 (0.27 - 0.44)	0.02 (0.01 - 0.05)
Quechua	0.03 (0.01 - 0.05)	0.72 (0.63 - 0.80)	0.01 (0.00 - 0.03)
Samburu	0.05 (0.03 - 0.08)	0.53 (0.44 - 0.61)	0.03 (0.01 - 0.06)
Wichi	0.04 (0.02 - 0.07)	0.61 (0.52 - 0.71)	0.01 (0.00 - 0.04)

Marbles marginalising out development

```r
margdev_preds %>%
    filter(accident_type == "marbles") %>%
    pivot_wider(id_cols = "culture", names_from = "helping_type", values_from= "sumstr") %>%
    arrange(culture) %>%
    kable()
```

culture	info	inst	both
German	0.04 (0.02 - 0.07)	0.53 (0.45 - 0.61)	0.04 (0.01 - 0.09)
Haikom	0.04 (0.02 - 0.06)	0.64 (0.56 - 0.73)	0.02 (0.01 - 0.04)
Kikuyu	0.07 (0.04 - 0.10)	0.36 (0.28 - 0.44)	0.02 (0.01 - 0.05)
Quechua	0.03 (0.02 - 0.05)	0.71 (0.63 - 0.79)	0.01 (0.01 - 0.03)
Samburu	0.05 (0.03 - 0.08)	0.52 (0.44 - 0.60)	0.03 (0.01 - 0.06)
Wichi	0.05 (0.03 - 0.09)	0.60 (0.51 - 0.69)	0.01 (0.00 - 0.04)

Developmental trends

```r
nd <- expand_grid(z.age=seq(-1, 1, 0.1), z.sex=c(-0.5, 0.5), z.accident_type=c(-0.5, 0.5), culture=uniq
int_dev_preds <- avg_predictions(m, newdata=nd, by=c("culture", "z.accident_type", "z.sex", "z.age")) %
    as_tibble() %>%
    tidy_labels() %>%
    mutate(help_type = case_when(group == 1 ~ "no help",
                     group == 2 ~ "informational",
                     group == 3 ~ "instrumental",
                     group == 4 ~ "both together"))

int_dev_preds %>%
    mutate(help_type = factor(help_type, c("no help", "informational", "instrumental", "both together"))
    ggplot() +
    geom_line(aes(x=age, y=estimate, colour=culture, linetype=sex)) +
    facet_grid(accident_type ~ help_type)
```

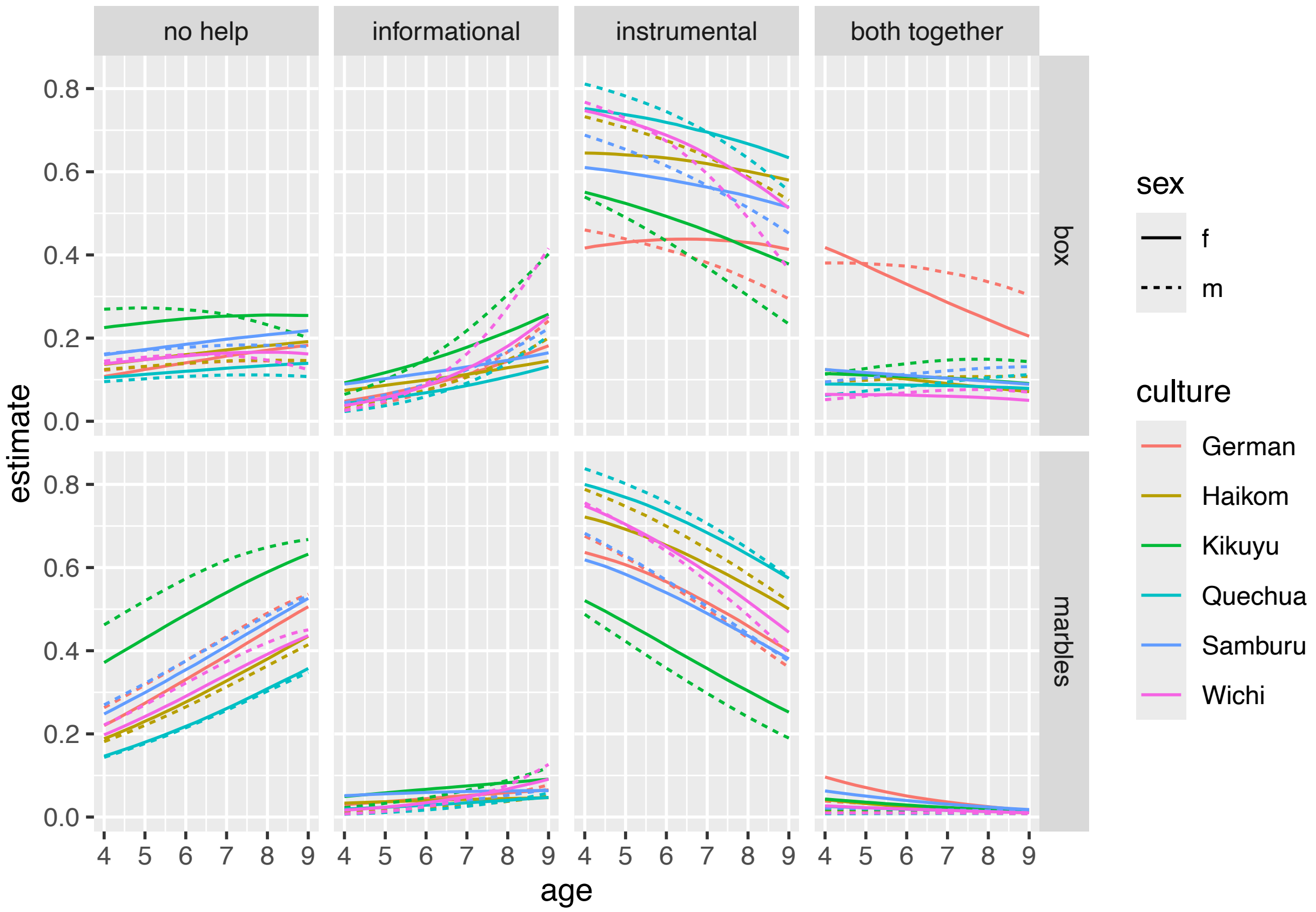

Helping in response to marble actions was almost always of the instrumental variety. The probability of providing instrumental help decreased with age, while the probability of not providing any help increased correspondingly. Providing informational help or informational and instrumental help together was very rare across the full age range in all cultures.

The developmental trajectory for helping in response to box accidents was more complicated. While the probability of offering some kind of help remained relatively constant across the full age range in all cultures, the relative likelihood of the different helping behaviours showed some change. Generally speaking, young children were much more likely to offer instrumental help alone. Children gradually became less likely to offer instrumental help and more likely to offer informational help. The extent of this shift in preference varied; in some demographics, the oldest children were roughly equally likely to offer informational or instrumental help, but in most cases instrumental help remained more likely. The German participants were an exception to this trend; young Germans were roughly equally likely to provide instrumental help alone or in combination with informational help. The tendency to offer both kinds of help simultaneously decreased slightly with age, but remained more likely than offering informational alone. This is one of the starkest cross-cultral differences in the dataset: German children responding to box accidents were between 3 to 4 times more likely to offer both kinds of help together than children from any other country. There is also evidence of another cross-cultural difference in developmental trends: the posterior probability that Wichi children increase their probability of information-only reponses with age at a rate above the cross-cultural mean is 0.95.

Sex differences

Our data does not show very strong evidence for direct effects of sex, with none of the 95% HPD intervals for the sex predictors excluding zero. There is moderate to strong evidence for sex interacting with some other predictors, though.

For example, averaging across accident types, providing informational help only becomes more probable with

increasing age. The posterior probability that this increase happens faster in boys than girls is 0.90: the 95% HPD interval of the age slope for boys entirely excludes zero. For girls, the 95% HPD interval includes zero, but a positive age slope is twice as credible as as a negative slope.

The probability of providing informational help only, as well as that of providing both informational and instrumental help together, both vary not only with age, but with accident type, as noted previously. The posterior probabilities that these effects of accident type varies between sexes are 0.72 (information only) and 0.85 (both together). However, the magnitude of these sex interaction is very small relative to the main effect of accident type. Additionally, providing instrumental help is generally much more frequent than either of these other possibilities, and we find almost no evidence for an interaction between sex and accident type for this behaviour (posterior mean interaction 0.09, 95% HPD interval -0.47 - 0.67).

We therefore conclude that while some differences between boys and girls appear quite plausible, the type of accident, the age of the child and variation across cultures appear to have much more influence on helping behaviour.

Full model summary

```
summary(m)
```

```
##  Family: categorical
##   Links: mu2 = logit; mu3 = logit; mu4 = logit
## Formula: help_cat ~ 1 + z.accident_type + z.age + z.sex + z.accident_type:z.age + z.accident_type:z.s
##    Data: d (Number of observations: 1133)
##   Draws: 4 chains, each with iter = 2000; warmup = 1000; thin = 1;
##          total post-warmup draws = 4000
##
## Multilevel Hyperparameters:
## ~culture (Number of levels: 6)
##                                          Estimate Est.Error l-95% CI u-95% CI
## sd(mu2_Intercept)                            0.11      0.10     0.00     0.38
## sd(mu2_z.accident_type)                      0.18      0.17     0.00     0.58
## sd(mu2_z.age)                                0.35      0.23     0.01     0.87
## sd(mu2_z.sex)                                0.21      0.18     0.01     0.66
## sd(mu3_Intercept)                            0.49      0.15     0.26     0.84
## sd(mu3_z.accident_type)                      0.20      0.15     0.01     0.54
## sd(mu3_z.age)                                0.09      0.09     0.00     0.32
## sd(mu3_z.sex)                                0.22      0.15     0.01     0.57
## sd(mu4_Intercept)                            0.47      0.20     0.10     0.91
## sd(mu4_z.accident_type)                      0.46      0.30     0.02     1.12
## sd(mu4_z.age)                                0.26      0.19     0.01     0.71
## sd(mu4_z.sex)                                0.16      0.16     0.00     0.56
## cor(mu2_Intercept,mu2_z.accident_type)      -0.04      0.45    -0.84     0.81
## cor(mu2_Intercept,mu2_z.age)                 0.04      0.44    -0.79     0.82
## cor(mu2_z.accident_type,mu2_z.age)           0.07      0.44    -0.78     0.84
## cor(mu2_Intercept,mu2_z.sex)                 0.04      0.44    -0.80     0.82
## cor(mu2_z.accident_type,mu2_z.sex)           0.02      0.44    -0.81     0.83
## cor(mu2_z.age,mu2_z.sex)                     0.14      0.44    -0.75     0.86
## cor(mu3_Intercept,mu3_z.accident_type)      -0.24      0.40    -0.88     0.64
## cor(mu3_Intercept,mu3_z.age)                 0.03      0.43    -0.78     0.80
## cor(mu3_z.accident_type,mu3_z.age)          -0.01      0.44    -0.81     0.80
## cor(mu3_Intercept,mu3_z.sex)                 0.29      0.39    -0.57     0.88
## cor(mu3_z.accident_type,mu3_z.sex)          -0.14      0.43    -0.86     0.72
## cor(mu3_z.age,mu3_z.sex)                     0.06      0.44    -0.78     0.82
```

```
## cor(mu4_Intercept,mu4_z.accident_type)     0.25      0.38    -0.55     0.86
## cor(mu4_Intercept,mu4_z.age)              -0.27      0.40    -0.89     0.58
## cor(mu4_z.accident_type,mu4_z.age)        -0.15      0.42    -0.86     0.70
## cor(mu4_Intercept,mu4_z.sex)              -0.01      0.44    -0.80     0.78
## cor(mu4_z.accident_type,mu4_z.sex)         0.02      0.44    -0.80     0.81
## cor(mu4_z.age,mu4_z.sex)                  -0.03      0.44    -0.82     0.80
##                                         Rhat Bulk_ESS Tail_ESS
## sd(mu2_Intercept)                       1.00     3276     2263
## sd(mu2_z.accident_type)                 1.00     2989     2175
## sd(mu2_z.age)                           1.00     1623     1923
## sd(mu2_z.sex)                           1.00     2874     2064
## sd(mu3_Intercept)                       1.00     2480     2586
## sd(mu3_z.accident_type)                 1.00     2012     2051
## sd(mu3_z.age)                           1.00     3086     2628
## sd(mu3_z.sex)                           1.00     2081     2057
## sd(mu4_Intercept)                       1.00     1727      804
## sd(mu4_z.accident_type)                 1.00     1722     1787
## sd(mu4_z.age)                           1.00     2261     2230
## sd(mu4_z.sex)                           1.00     3467     2474
## cor(mu2_Intercept,mu2_z.accident_type) 1.00     6341     2739
## cor(mu2_Intercept,mu2_z.age)            1.00     3136     3108
## cor(mu2_z.accident_type,mu2_z.age)      1.00     3056     3082
## cor(mu2_Intercept,mu2_z.sex)            1.00     5973     2753
## cor(mu2_z.accident_type,mu2_z.sex)      1.00     3727     3087
## cor(mu2_z.age,mu2_z.sex)                1.00     3449     3214
## cor(mu3_Intercept,mu3_z.accident_type) 1.00     4816     2623
## cor(mu3_Intercept,mu3_z.age)            1.00     6579     2807
## cor(mu3_z.accident_type,mu3_z.age)      1.00     5213     3026
## cor(mu3_Intercept,mu3_z.sex)            1.00     5290     3038
## cor(mu3_z.accident_type,mu3_z.sex)      1.00     4031     3298
## cor(mu3_z.age,mu3_z.sex)                1.00     3263     3425
## cor(mu4_Intercept,mu4_z.accident_type) 1.00     4283     2186
## cor(mu4_Intercept,mu4_z.age)            1.00     4661     3014
## cor(mu4_z.accident_type,mu4_z.age)      1.00     3561     2954
## cor(mu4_Intercept,mu4_z.sex)            1.01     7534     2937
## cor(mu4_z.accident_type,mu4_z.sex)      1.00     4698     2651
## cor(mu4_z.age,mu4_z.sex)                1.00     4241     3155
##
## Regression Coefficients:
##                           Estimate Est.Error l-95% CI u-95% CI Rhat Bulk_ESS
## mu2_Intercept                -1.33      0.16    -1.65    -1.03 1.00     5317
## mu3_Intercept                 0.84      0.22     0.39     1.28 1.00     1805
## mu4_Intercept                -1.64      0.28    -2.22    -1.10 1.00     2728
## mu2_z.accident_type           1.78      0.30     1.21     2.38 1.00     4807
## mu2_z.age                     0.51      0.27    -0.00     1.08 1.00     4190
## mu2_z.sex                    -0.21      0.30    -0.79     0.38 1.00     4456
## mu2_z.accident_type:z.age     0.42      0.42    -0.41     1.22 1.00     5533
## mu2_z.accident_type:z.sex     0.47      0.53    -0.58     1.50 1.00     6014
## mu2_z.age:z.sex               0.61      0.39    -0.16     1.40 1.00     6103
## mu3_z.accident_type           0.77      0.19     0.39     1.14 1.00     4067
## mu3_z.age                    -0.45      0.13    -0.70    -0.20 1.00     5644
## mu3_z.sex                    -0.02      0.19    -0.40     0.36 1.00     3872
## mu3_z.accident_type:z.age     0.38      0.23    -0.07     0.83 1.00     5962
## mu3_z.accident_type:z.sex     0.09      0.29    -0.47     0.67 1.00     5721
```

```
## mu3_z.age:z.sex            -0.02      0.22    -0.45     0.42 1.00     6148
## mu4_z.accident_type         2.60      0.42     1.79     3.46 1.00     3835
## mu4_z.age                  -0.44      0.28    -1.00     0.13 1.00     3574
## mu4_z.sex                  -0.23      0.35    -0.94     0.44 1.00     4627
## mu4_z.accident_type:z.age   0.68      0.49    -0.28     1.66 1.00     4199
## mu4_z.accident_type:z.sex   0.87      0.63    -0.37     2.11 1.00     4846
## mu4_z.age:z.sex             0.44      0.38    -0.31     1.18 1.00     6325
##                         Tail_ESS
## mu2_Intercept               3411
## mu3_Intercept               2276
## mu4_Intercept               2813
## mu2_z.accident_type         2940
## mu2_z.age                   2976
## mu2_z.sex                   3065
## mu2_z.accident_type:z.age   3490
## mu2_z.accident_type:z.sex   2848
## mu2_z.age:z.sex             3269
## mu3_z.accident_type         2884
## mu3_z.age                   3136
## mu3_z.sex                   2646
## mu3_z.accident_type:z.age   3254
## mu3_z.accident_type:z.sex   3132
## mu3_z.age:z.sex             3065
## mu4_z.accident_type         2556
## mu4_z.age                   2923
## mu4_z.sex                   2992
## mu4_z.accident_type:z.age   2872
## mu4_z.accident_type:z.sex   2968
## mu4_z.age:z.sex             3719
##
## Draws were sampled using sampling(NUTS). For each parameter, Bulk_ESS
## and Tail_ESS are effective sample size measures, and Rhat is the potential
## scale reduction factor on split chains (at convergence, Rhat = 1).
```

2 References

1. U. Dieckmann, Scraping the pot: San in Namibia two decades after independence. (2014).

2. T. Widlok, Living On Mangetti:'Bushman'Autonomy and Namibian Independence. (2001).

3. M. Schäfer, D. B. Haun, M. Tomasello, Fair is not fair everywhere. *Psychol. Sci.* **26**, 1252–1260 (2015).

4. S. Lew-Levy, N. Lavi, R. Reckin, J. Cristóbal-Azkarate, K. Ellis-Davies, How do hunter-gatherer children learn social and gender norms? A meta-ethnographic review. *Cross-Cult. Res.* **52**, 213–255 (2018).

5. H. Terashima, B. S. Hewlett, *Social learning and innovation in contemporary hunter-gatherers* (Springer, 2016).

6. J. Kenyatta, *Facing Mt. Kenya: The Tribul Life of the Gikuyu* (Secker & Warburg, 1938).

7. B. B. Whiting, The effect of social change on concepts of the good child and good mothering: A study of families in Kenya. *Ethos* **24**, 3–35 (1996).

8. N. Price, The changing value of children among the Kikuyu of Central Province, Kenya. *Africa* **66**, 411–436 (1996).

9. S. Leipzig, Einwohnerzahl und Bevölkerungsentwicklung in Leipzig. *Pobrano Z Httpswww Leipz. Debuergerservice--Verwaltungunsere-Stadtstatistik-Undzahleneinwohner--Bevoelkerungsentwicklung* (2021).

10. E. S. Durgel, B. Leyendecker, B. Yagmurlu, R. Harwood, Sociocultural influences on German and Turkish immigrant mothers' long-term socialization goals. *J. Cross-Cult. Psychol.* **40**, 834–852 (2009).

11. E. P. de Bolivia, Censo de Población y Vivienda 2012 Bolivia. Características de la Población. (2015).

12. M. Walsh-Dilley, Theorizing reciprocity: Andean cooperation and the reproduction of community in highland Bolivia. *J. Lat. Am. Caribb. Anthropol.* **22**, 514–535 (2017).

13. P. P. Martínez, Why Nazario is leaving school: Community perspectiveson formal schooling in rural Bolivia. *Knowl. Learn. Andes Ethnogr. Perspect.* 141–165 (2002).

14. P. Spencer, *The Samburu: A study in geocentracy* (Routledge, 2004).

15. E. S. Miller, Peoples of the Gran Chaco. *No Title* (1999).

16. I. Argentina, D. Empleados, Y. DE COMERCIO, Censo Nacional de Población, Hogares y Viviendas 2010. *Recuperado Httpswww Indec Gob ArindecwebNivel4-CensoNacional-999-999-Censo-2010* (2010).

17. J. Palmer, La buena voluntad Wichí: una espiritualidad indígena. Formosa, Salta. *APCDCECAZOEPRAZOL Grupo Trab. Ruta 81 2005 432 P Ill Dispon. En Httpsdoi Org104000jsa 8423* (2005).

18. A. S. Taverna, S. R. Waxman, D. L. Medin, N. Moscoloni, O. A. Peralta, Naming the living things: Linguistic, experiential and cultural factors in Wichí and Spanish speaking children. *J. Cogn. Cult.* **14**, 213–233 (2014).

19. J. J. Rossi, *Los Wichí (los" Mataco")* (Editorial Galerna, 2003).

20. J. R. Landis, G. G. Koch, The measurement of observer agreement for categorical data. *biometrics* 159–174 (1977).

21. P.-C. Bürkner, brms: An R package for Bayesian multilevel models using Stan. *J. Stat. Softw.* **80**, 1–28 (2017).
